# Predictive modelling of response to neoadjuvant therapy in HER2+ breast cancer

**DOI:** 10.1038/s41523-023-00572-9

**Published:** 2023-09-27

**Authors:** Nicola Cosgrove, Alex J. Eustace, Peter O’Donovan, Stephen F. Madden, Bruce Moran, John Crown, Brian Moulton, Patrick G. Morris, Liam Grogan, Oscar Breathnach, Colm Power, Michael Allen, Janice M. Walshe, Arnold D. Hill, Anna Blümel, Darren O’Connor, Sudipto Das, Małgorzata Milewska, Joanna Fay, Elaine Kay, Sinead Toomey, Bryan T. Hennessy, Simon J. Furney

**Affiliations:** 1 Genomic Oncology Research Group, Department of Physiology and Medical Physics, RCSI University of Medicine and Health Sciences, Dublin, Ireland; 2 School of Biotechnology, National Institute for Cellular Biotechnology, Dublin City University, Dublin, Ireland; 3 Data Science Centre, RCSI University of Medicine and Health Sciences, Dublin, Ireland; 4 Conway Institute, University College Dublin, Dublin, Ireland; 5 Department of Medical Oncology, St Vincent’s University Hospital, Dublin, Ireland; 6 Clinical Oncology Development Europe, Dublin, Ireland; 7 Department of Medical Oncology, Beaumont Hospital, Dublin, Ireland; 8 Department of Surgery, RCSI University of Medicine and Health Sciences, Dublin, Ireland; 9 School of Pharmacy and Biomolecular Sciences, RCSI University of Medicine and Health Sciences, Dublin, Ireland; 10 Medical Oncology Group, Department of Molecular Medicine, Royal College of Surgeons in Ireland, Dublin, 9 Ireland; 11 RCSI Biobank Service, RCSI University of Medicine and Health Sciences, Beaumont Hospital, Dublin, 9 Ireland; 12 Department of Pathology, RCSI University of Medicine and Health Sciences, Beaumont Hospital, Dublin, 9 Ireland

**Keywords:** Breast cancer, Cancer genomics

## Abstract

HER2-positive (HER2+) breast cancer accounts for 20–25% of all breast cancers. Predictive biomarkers of neoadjuvant therapy response are needed to better identify patients with early stage disease who may benefit from tailored treatments in the adjuvant setting. As part of the TCHL phase-II clinical trial (ICORG10–05/NCT01485926) whole exome DNA sequencing was carried out on normal-tumour pairs collected from 22 patients. Here we report predictive modelling of neoadjuvant therapy response using clinicopathological and genomic features of pre-treatment tumour biopsies identified age, estrogen receptor (ER) status and level of immune cell infiltration may together be important for predicting response. Clonal evolution analysis of longitudinally collected tumour samples show subclonal diversity and dynamics are evident with potential therapy resistant subclones detected. The sources of greater pre-treatment immunogenicity associated with a pathological complete response is largely unexplored in HER2+ tumours. However, here we point to the possibility of APOBEC associated mutagenesis, specifically in the ER-neg/HER2+ subtype as a potential mediator of this immunogenic phenotype.

## Introduction

Human epidermal growth factor receptor 2 (HER2) positive breast cancer driven by HER2 gene amplification or protein overexpression accounts for 20–25% of invasive breast cancers^1^. Neoadjuvant anti-HER2 targeted therapy in combination with chemotherapy is the standard of care for patients with HER2+ early stage breast cancer regardless of estrogen receptor (ER) status^2,3^. Despite a substantial improvement in terms of overall and disease-free survival for HER2+ patients since the addition of anti HER2 targeted therapy to chemotherapy in the neoadjuvant setting^4^, up to ~30% of patients will develop treatment resistance where disease progression and metastases is of concern^5^. Pathologic assessment of the breast and axillary nodes is used to assess the presence and extent of residual invasive disease (RD) following neoadjuvant treatment. Pathologic complete response (pCR) is defined as absence of invasive tumour in breast and lymph node (ypT0/is; ypN0)^6^. Response to neoadjuvant therapy is a surrogate marker of patient prognosis^5,7^, where pCR is associated with improved disease-free and overall survival^7,8^.

Many clinical trials in recent years have investigated if the addition of other HER-directed therapies such as the TKI lapatinib to neoadjuvant trastuzumab plus chemotherapy^9–12^ could lead to improved pCR rates^13^. One such clinical trial (ICORG10–05/NCT01485926) was a phase II neoadjuvant study assessing TCH (Docetaxel, Carboplatin and Trastuzumab) and TCHL (Docetaxel, Carboplatin, Trastuzumab and Lapatinib) in early stage HER2+ breast cancer. One of the secondary objectives of the trial was to identify biomarkers of anti-HER2 therapy response or resistance^14^. In early stage HER2+ breast cancer many studies have reported on features associated with attainment of pathologic complete response to neoadjuvant therapy^14–18^. Previous work characterising the genome of neoadjuvant treatment HER2+ breast cancer has demonstrated that mutations in known cancer driver genes such as PIK3CA^14,18,19^, the immune response^20^ and the HER2-enriched mRNA intrinsic molecular subtype may all have a role in predicting treatment response^5,11,15,17,21^. Overall these studies have largely focused on analysis of pre-treatment tumour biopsies alone. Few studies^17^ have used longitudinally collected samples for temporal dissection of the genomic changes that may occur during the evolution of neoadjuvant treated HER2+ breast cancer tumours. Clonal evolution analysis may help elucidate characteristics of tumours that do not respond to treatment and as such may have residual disease at surgery.

Here, in order to identify which clinicopathological and genomic features may be predictive of neoadjuvant therapy response, whole exome DNA sequencing (WXS) was performed on normal-tumour sample pairs collected from 22 of 88 cases who took part in the TCHL phase-II clinical trial. Recurrent somatic copy number alterations (SCNA), single nucleotide variants (SNVs), InDels, mutational signatures and estimated T cell fraction were identified from analysis of WXS data. Clinicopathological and genomic features extracted from pre-treatment tumour biopsy data were used as input to a series of predictive models for predicting future pathological complete response. The predictive models were trained and tested in independent, external cohorts of patients with early stage HER2+ breast cancer. Validation of model accuracy was performed in the HER2 + TCHL cohort presented here. From this, we identified a set of features which together are important for predicting pCR. Furthermore, tumour evolutionary analysis was carried out for 5 / 22 cases for which high depth WXS was available from samples taken at multiple timepoints during the course of treatment. In-depth genomic characterization of pre-treatment biopsies and later timepoints in early stage HER2+ breast cancer together with predictive modelling has the potential to identify biomarkers of neoadjuvant therapy tumour response.

## Results

### Clinical characteristics of HER2+ whole exome DNA sequencing cohort

**Fig. 1:**
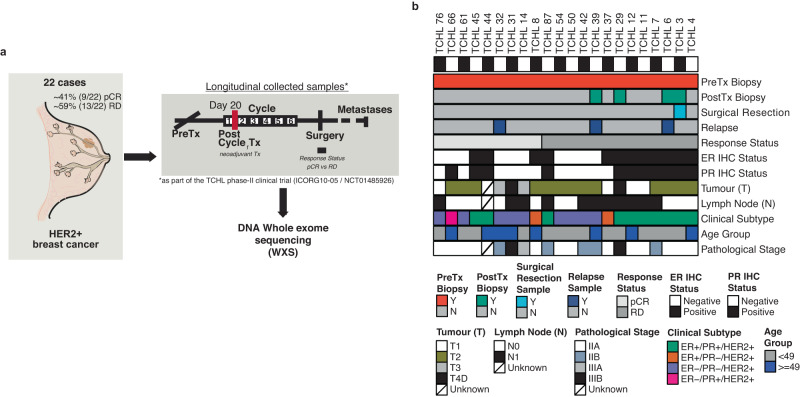
Overview of the HER2 + TCHL WXS cohort. **a** Graphical overview of the HER2 + TCHL whole exome sequencing (WXS) breast cancer cohort (22 cases). Text annotation details the percentage (%) and number of the 22 cases which had either pathological complete response (pCR) or residual disease (RD) in response to neoadjuvant treatment (left). Longitudinal collected samples collected as part of the TCHL phase II clinical trial included pre-treatment (PreTx), Post treatment (PostTx) cycle 1 (Day 20) tumour biopsies, surgical resection and if occurred, metastatic tumour samples. **b** Tileplot outlines for each HER2+ breast cancer case in the TCHL WXS cohort (left-right) the type of tumour sample collected and clinicopathological characteristics (top-bottom). ER estrogen receptor, IHC immunohistochemistry, PR progesterone receptor.

WXS was performed on normal-tumour sample pairs collected from twenty-two patients with HER2+ early stage breast cancer who had received neoadjuvant anti-HER2 targeted therapy in combination with chemotherapy as part of the TCHL phase II clinical trial (ICORG10–05/NCT01485926) (Fig. 1a). Tumour samples included twenty-two pre-treatment core biopsies and for 5 of 22 cases longitudinally collected samples including 4 post-treatment cycle one (Day-20) biopsies, 1 surgical resection sample and 3 metastatic tumours (Fig. 1b; Supplementary Table 1). Evaluation of tumour response status at primary surgery identified ~41% (*n* = 9) had attained a pathological complete response (pCR) while ~59% (*n* = 13) were non-pCR having residual disease (RD) (Table 1). Clinicopathological characteristics of this cohort included median age at diagnosis of 49 years [40,79] and 45 years [34,69] for those who had a pathological complete response or residual disease respectively. Hormone receptor status of the primary tumour at diagnosis included 12 ER-positive and 10 ER-negative cases. Of note, ~69% (9 of 13 cases) with RD were ER-positive, consistent with other studies where has been observed rates of pCR in neoadjuvant treated HER2+ breast cancer are lower in patients with ER-positive tumours^5,7,11,17^.

**Table 1:** Clinical characteristics of the HER2+ neoadjuvant treated cohort stratified by tumour response status.

	pCR	RD
No. of samples (%)	9 (40.9)	13 (59.1)
Age at diagnosis*	49 [40, 79]	45 [34, 69]
Overall Stage (%)		
II	6 (75.0)	12 (92.3)
III	2 (25.0)	1 (7.7)
ER Status by IHC (%)		
Positive (+)	3 (33.3)	9 (69.2)
Negative (−)	6 (66.7)	4 (30.8)
PR Status by IHC (%)		
Positive (+)	3 (33.3)	8 (61.5)
Negative (−)	6 (66.7)	5 (38.5)
HER2 Status		
Positive (+)	9 (40.9)	13 (59.1)
Negative (−)	0	0
Clinical Subtype (%)		
ER+/PR+/HER2+	2 (22.2)	8 (61.5)
ER+/PR−/HER2+	1 (11.1)	1 (7.7)
ER−/PR−/HER2+	5 (55.6)	4 (30.8)
ER−/PR+/HER2+	1 (11.1)	0 (0.0)
Anti HER2 Targeted therapy (%)^†^		
Trastuzumab	4 (50.0)	3 (33.3)
Trastuzumab + Lapatinib	4 (50.0)	6 (66.7)

*pCR* pathological complete response, *RD* residual disease.

*Median [range].

^†^Samples for WXS collected as part of the ICORG10–05/NCT01485926 Phase II TCHL clinical trial TCH (Trastuzumab), TCHL (Trastuzumab+ Lapatinib).

### Somatic genomic alterations in pre-treatment tumour biopsies

**Fig. 2:**
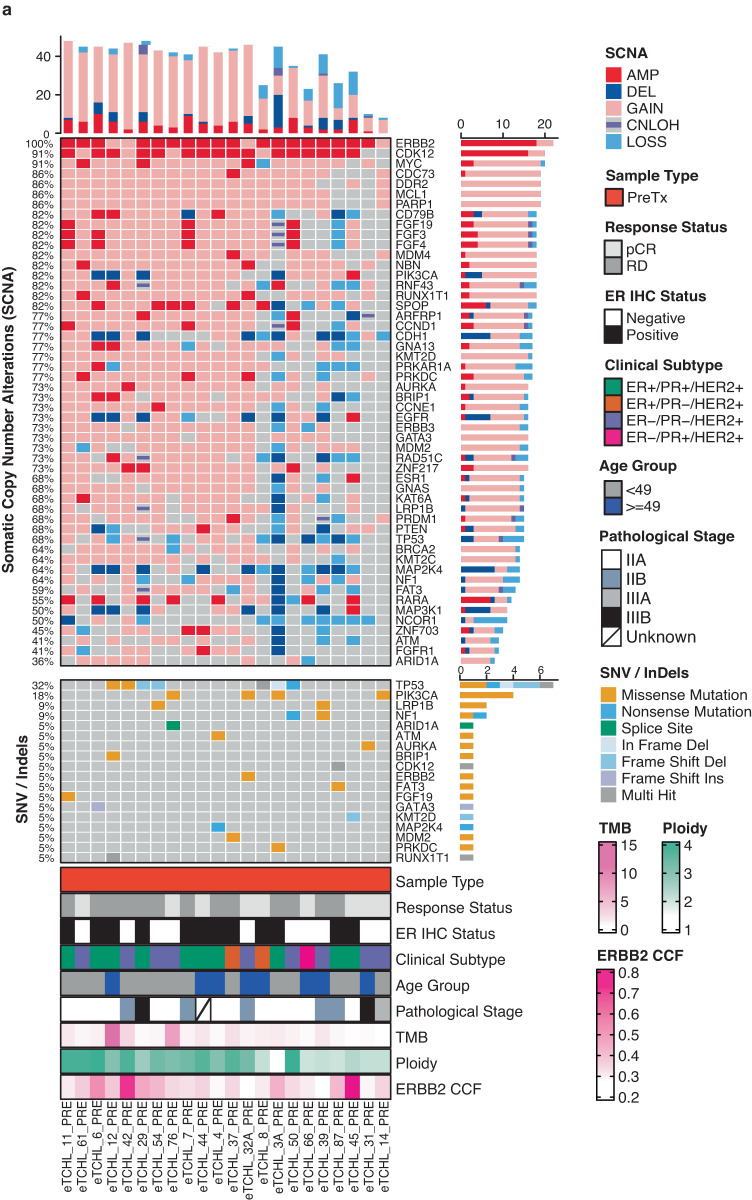
Somatic genomic alterations of HER2+ pre-treatment biopsies. **a** Oncoplot of recurrent somatic genomic alterations including somatic copy number alterations (SCNA) (top) and SNVs and InDels (middle) in known breast cancer driver genes frequently altered in HER2+ specific tumour subtype across HER2+ pre-treatment biopsies (*n* = 22) (left-right). Samples annotated (bottom) by clinico-pathological characteristics of the HER2+ neoadjuvant treated WXS cohort. Also annotated is tumour ploidy, ERBB2 (HER2) copy number cancer cell fraction (CCF) extracted from FACETS SCNA data for each tumour sample.

In order to characterise the pre-treatment somatic genomic alteration landscape of neoadjuvant treated tumours, somatic mutation and copy number calling was performed on normal-tumour sample pairs for all patients (Fig. 2a; Supplementary Fig. 1, 2). As expected HER2 (ERBB2) was the most frequently altered gene in this cohort with all cases having either a copy number amplification or gain in the HER2 (ERBB2) gene (Fig. 2a). We identified frequent copy number alterations, SNVs and InDels in other breast cancer driver genes previously reported to be frequently altered in HER2+ specific breast cancer (Supplementary Fig. 3; Supplementary Table 2–4) including CDK12, MYC, PIK3CA with copy number loss or somatic mutations in known tumour suppressor genes including TP53 and NF1.

### Characterisation of ERBB2 (HER2) and PI3K-AKT-mTOR pathway specific genomic alterations

**Fig. 3:**
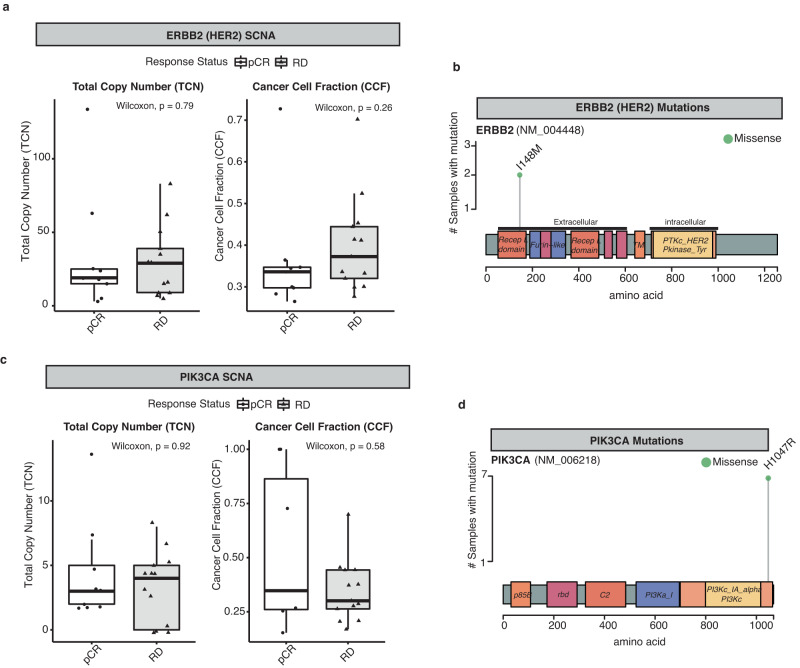
Characterisation of ERBB2 (HER2) and PIK3CA specific genomic alterations. **a** Boxplots show the distribution of total copy number (TCN) (left) and cancer cell fraction (CCF) (right) values for ERBB2 (HER2) SCNA in pCR (*n* = 9; white) versus RD (*n* = 13; grey) classified pre-treatment tumour samples (Wilcox Test, all not statistically significant (*P* > 0.05)). **b** Lolliplot of recurrent missense mutation L148M (green dot) identified in ERBB2 (HER2) protein from WXS data. **c** Boxplots show the distribution of total copy number (TCN) (left) and cancer cell fraction (ccf) (right) values for PIK3CA SCNA in pCR (*n* = 9; white) versus RD (*n* = 13; grey) classified pre-treatment tumour samples (Wilcox Test, all not statistically significant (*P* > 0.05)). **d** Lolliplot of recurrent missense mutation H1047R (green dot) identified in PIK3CA protein from WXS data. Horizontal lines in the box plots denote the lower quartile (Q1), median and upper quartile (Q3). The box bounds the interquartile range (IQR = Q3−Q1) with the whiskers denoting 1.5 × IQR. Wilcoxon rank sum tests, all *P* values two-sided.

Dysregulated PI3K-AKT-mTOR pathway signaling due to either activating somatic mutations in PIK3CA or reduced expression of the protein phosphatase tumour suppressor PTEN is often reported to be associated with development of anti-HER2 therapy resistance and may be predictive of pCR in HER2+ breast cancer^14,22,23^. As such we investigated HER2, PIK3CA and PTEN specific genomic alterations in this cohort in greater detail. There was no statistically significant difference in ERRB2 (HER2) total gene copy number (pCR:19 and RD:29 median total copies; *P* = 0.79) and ERBB2 (HER2) cancer cell fraction (pCR: 0.33 and RD: 0.37 median CCF; *P* = 0.26) when comparing pCR (*n* = 9) to RD (*n* = 13) groups (Fig. 3a; Wilcox Test, *P* > 0.05; Supplementary Table 5). A subclonal ERBB2 (HER2) somatic missense mutation (p.I148M) was detected in the receptor ligand domain for both the pre-treatment tumour biopsy (~0.12 VAF) and patient matched brain metastatic tumour (~0.08 VAF) for Case #32 (Fig. 3b; Supplementary Table 6). Its functional relevance is uncertain given that ERBB2 (HER2) receptor activity is independent of bound ligand.

For the PIK3CA gene, somatic copy number alterations in pre-treatment tumour biopsies included copy number gain in ~45% (10/22) or amplification in ~18% (4/22) of HER2+ breast cancer cases. We identified in 4 cases (7 tumour samples: 4 pre-treatment biopsies, 1 post cycle 1 treatment biopsy and 1 metastatic tumour) an exon 20 somatic missense mutation p.H1047R (Fig. 3d; Supplementary Table 6). Here, 2 of 4 cases with a PIK3CA p.H1074R mutation had a pCR (both ER+), with the other two cases having residual disease (1 ER+, 1 ER−). Interestingly, PIK3CA somatic copy number alterations were overall mutually exclusive of PIK3CA H1047R mutation. There was no statistically significant difference in PIK3CA gene total copy number (*P* = 0.92) between pCR (*n* = 9) and RD (*n* = 13) groups (Fig. 3c; Wilcox Test, *P* > 0.05). We did note however, numerical higher median PIK3CA copy number in pre-treatment biopsies which went on to have residual disease (*P* = 0.92) with the exception of one case with PIK3CA amplification (total copy number > 10) which had a pCR (Fig. 3c). For PTEN, copy number deletion or loss was identified in 4 of 22 pre-treatment tumour biopsies with all having residual disease at surgery (Fig. 2a). Interestingly, in one case where we identified a PTEN copy number amplification, this patient had attained a pCR, suggesting high levels of PTEN may have contributed to tumour regression^24^.

### Mutational signature profiles of HER2+ pre-treatment biopsies

**Fig. 4:**
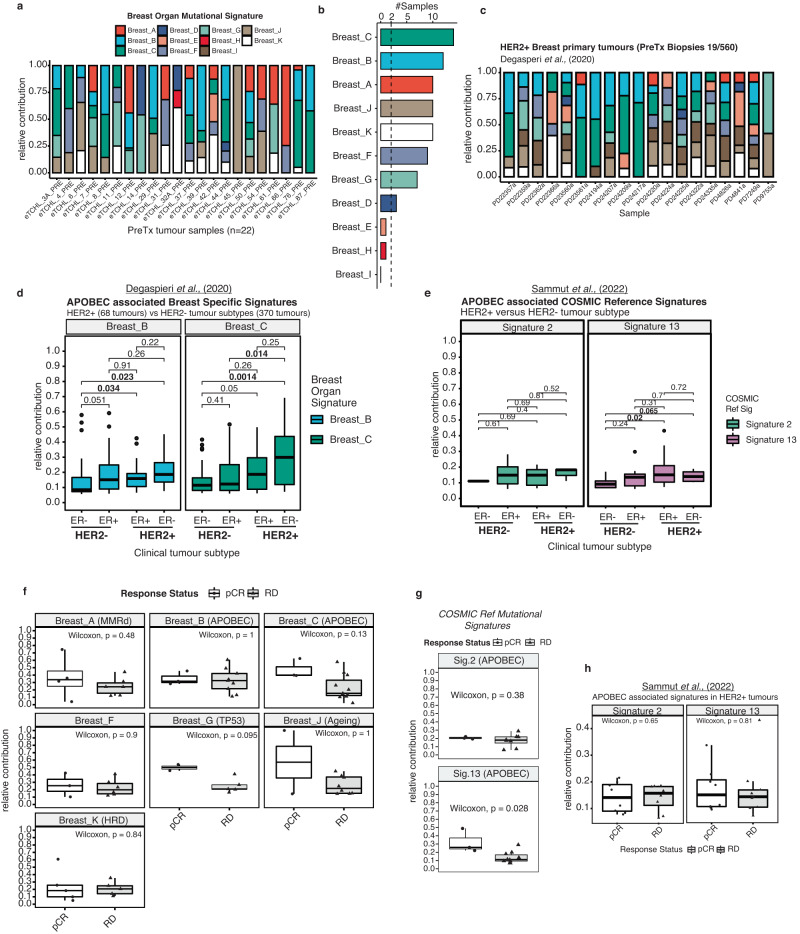
Mutational signature profiles of HER2+ pre-treatment biopsies. **a** Stacked barchart of the relative contribution [0–1] of breast cancer specific reference mutational signatures (Breast *A-K*) detected in each pre-treatment tumour biopsy sample (left-right) from the HER2 + TCHL WXS cohort. **b** Barchart shows frequency of breast organ specific mutational signatures (top-bottom) across all pre-treatment biopsy samples (*n* = 22). **c** Stacked barchart of the relative contribution [0–1] of breast cancer specific reference mutational signatures (Breast *A-K*) detected in each pre-treatment biopsy tumour sample (left-right) from Degasperi et al., (2020) HER2+ breast cancer cohort (*n* = 19 tumours). **d** Boxplots show distribution of APOBEC associated mutational signature (Breast B (blue), C (green)) values stratified by clinical tumour subtype in Degasperi et al., (2020) breast cancer cohort (438 total tumours) (Wilcox Test; Tumour subtype pairwise comparisons *P*-values text annotation. Highlighted in bold text is statistically significant pairwise comparisons *P* < 0.05). **e** Boxplots show distribution of APOBEC associated mutational signature (COSMIC Reference Signature 2 (light green), Signature 13 (pink)) values stratified by clinical tumour subtype in Sammut et al., (2022) breast cancer cohort (Wilcox Test; Tumour subtype pairwise comparison *P*-values text annotation. Highlighted in bold text is statistically significant pairwise comparisons *P* < 0.05). **f** Boxplots show the distribution of relative contribution values in pre-treatment tumour biopsies (*n* = 22) of frequent breast organ specific reference signatures (Breast A-K) (present in >2 samples) in pCR (*n* = 9; white) compared to RD (*n* = 13; grey) classified tumours (Wilcox Test *P* < 0.05). **g** Boxplots show distribution of analogous APOBEC associated mutational signatures (Sig.2 (*P* = 0.38), Sig.13 (*P* = 0.028)) using deconstructSigs and COSMIC reference signatures in pCR (white) compared to RD (grey) classified tumours (Wilcox Test *P* < 0.05). **h** Boxplots show distribution of APOBEC associated mutational signature (COSMIC Reference Signature 2 (light green), Signature 13 (pink)) values stratified by tumour response status (pCR vs RD) in HER2+ only tumours in Sammut et al., (2022) breast cancer cohort (57 tumour samples) (Wilcox Test, *P* > 0.05). Horizontal lines in the box plots denote the lower quartile (Q1), median and upper quartile (Q3). The box bounds the interquartile range (IQR = Q3−Q1) with the whiskers denoting 1.5 × IQR. Wilcoxon rank sum tests, all P values two-sided.

Next, we investigated which mutational processes may be operational in pre-treatment biopsies. To perform mutational signature analysis we utilised catalogues of both somatic passenger and driver SNVs for each tumour (*n* = 22 samples) (See Methods). Mutational signatures extracted from pre-treatment biopsies were fitted to breast cancer specific signatures (Breast *A-K*) (Fig. 4a; Supplementary Table 7.1). Overall the most frequent mutational signatures detected from pre-treatment tumour biopsy data (*n* = 22), in descending order, were Breast *C* (*n* = 14) and Breast *B* (*n* = 12) (both APOBEC mutagenesis associated), Breast *A* (*n* = 10) (mismatch repair deficiency (MMRd)), Breast *J* (*n* = 10) (Ageing associated), Breast *K* (*n* = 10)(homologous recombination repair deficiency (HRD)), Breast *F* (*n* = 9) (aetiology unknown) and Breast *G* (*n* = 7) (TP53 mutation) (Fig. 4a). Less frequent (<=3 of 22 cases) signatures identified were Breast *D* (MMRd), Breast *E* (aetiology unknown) and Breast *H* (Fig. 4b; Supplementary Table 7.1). To confirm these findings we applied deconstructSigs, a method validated for whole exome sequencing data to fit COSMIC reference mutational signatures to the same set of tumour samples (Supplementary Fig. 4a–c; Supplementary Table 7.2). For the top five frequently detected mutational signatures in this cohort: Breast *C*, Breast *B*, Breast *A*, Breast *J*, Breast *K* (analogous to COSMIC reference *Sig.13, Sig.2, Sig.6, Sig.1* and *Sig.3)*, the overall percent (%) sample agreement between Signal breast organ specific signature detection and analogous COSMIC reference signature detection using deconstructSigs was ~95%, ~86%, ~59%, ~64% and ~73% respectively (Supplementary Table 7.2). Mutational signature profiles from a previously published primary breast cancer study^25,26^, which included some pre-treatment HER2+ tumour biopsies (n = 19), were overall similar in composition and sample frequency to the study cohort described here except for the presence of mutational signature Breast *I* (Fig. 4c; Supplementary Figs. 5, 6a). We prioritised APOBEC associated mutational signatures for further analysis given the frequency of the signatures in HER2+ pre-treatment tumours and having robustly confirmed detection across two methods and analogous mutational signature reference sets (Supplementary Table 7.2).

We first sought to assess if the APOBEC associated mutational signatures were more prevalent in the HER2+ versus HER2-negative tumour subtype overall. To address this, we utilised mutational signature profiles from previously published independent WXS datasets of primary breast cancer tumours (Supplementary Fig. 5). We observed that whilst the APOBEC associated mutational signatures Breast *B* and Breast *C* are present at some level in tumours across all breast cancer tumour subtypes, Breast *B (P* = 0.023*)* and Breast *C (P* = 0.0014*)* are significantly more prevalent in ER−/HER2+ versus ER−/HER2-neg tumour subtype (Fig. 4d; Supplementary Fig. 6b; ANOVA test followed by post-hoc pairwise Wilcox test). In an independent cohort enriched for pre-treated breast cancer tumours with WXS data and COSMIC reference mutational signature profiles (Sammut et al., (2022)), the APOBEC associated mutational signature Sig. 13 (analogous to Breast *C*) was significantly different between ER+/HER2+ versus ER-/HER2- tumour subtypes (Wilcox test *P* = 0.02). There was no statistically significant difference in the relative contribution of Sig. 13 (Breast *C*) between the ER−/ HER2+ versus ER−/HER2-neg in this cohort (Fig. 4e; Wilcox Test *P* = 0.065). Having observed greater prevalence of the APOBEC associated mutational signatures in HER2+ versus HER2− tumour subtypes, we next sought to assess which signatures be associated with future neoadjuvant therapy response. We compared the relative contribution of the Breast *A-K* signatures, if present (relative contribution > 0), in pre-treatment tumours from patients in the TCHL cohort which had attained a pCR to those with residual disease (Fig. 4f; Supplementary Fig. 7). We found no statistically significant difference in the relative contribution of each mutational signature when comparing pCR to RD groups (Fig. 4f). We noted however when performing the same test using COSMIC reference signature profile values instead, there was a statistically significant higher relative contribution of Sig. 13 (Wilcox test *P* = 0.028) (analogous to Breast *C*) in tumours which had attained a pCR (Fig. 4g; Supplementary Fig. 4d). However, this finding did not validate when testing APOBEC associated signatures in pre-treatment tumour biopsy samples from the external Sammut et al., (2022) (Fig. 4h; Supplementary Fig. 7) or the LeSurf et al., (2017) HER2+ breast cancer cohorts (Supplementary Fig. 8).

### Clinicopathological and genomic features associated with neoadjuvant treatment response

**Fig. 5:**
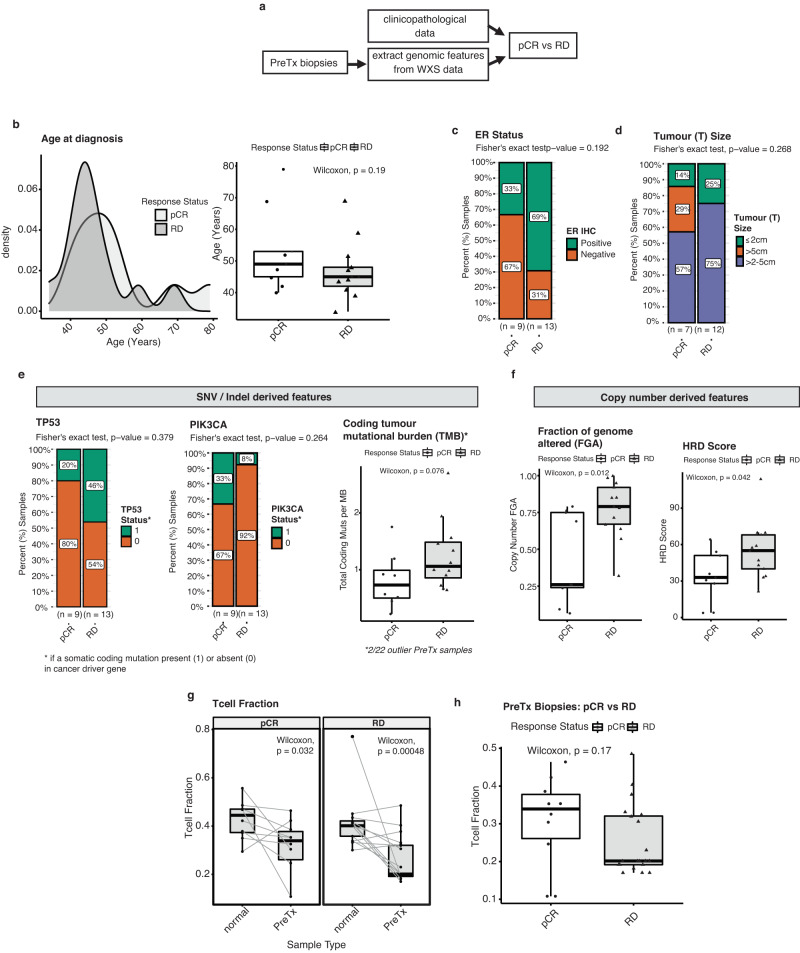
Clinicopathological and genomic features associated with neoadjuvant treated tumour response status (pathological complete response (pCR) vs residual disease (RD)). **a** Schematic diagram of genomic feature extraction from WXS data of pre-treatment tumour biopsies for pCR (*n* = 9) vs RD (*n* = 13) comparison. **b** Density plot (left) and boxplots (right) of age at diagnosis distribution for HER2+ cases (*N* = 22) stratified by tumour response status. **c** Stacked barchart of the proportion (%) of ER+ and ER-neg subtype tumours (**d**) Stacked barchart of the proportion (%) of tumour size (< = 2 cm, >2 cm–5 cm, > = 5 cm) derived from T Stage classification (**e**) Stacked barchart of the proportion (%) of pCR versus RD pre-treatment tumour biopsy samples with either presence (status = 1) or absence (status = 0) of TP53 or PIK3CA somatic coding mutation (left) (Fishers exact test, *P* > 0.05). Boxplots of coding tumour mutational burden (TMB) values in pCR versus RD pre-treatment tumour biopsies (19/22 samples; 2 extreme outlier samples removed)(Wilcox test *P* > 0.05). **f** Boxplots of the distribution of copy number derived feature values: fraction of genome altered (FGA) (left) and homologous recombination deficiency (HRD) score (right) in pCR versus RD pre-treatment tumour biopsies (Wilcox test *P* < 0.05). **g** Boxplots of the distribution of T cell fraction values in patient-matched normal with pre-treatment tumour biopsy sample stratified by tumour response status (Paired Wilcox test *P* < 0.05). **h** Boxplots of the distribution of T cell fraction values in pre-treatment tumour biopsies stratified by tumour response status (Wilcox test; *P* > 0.05). Horizontal lines in the box plots denote the lower quartile (Q1), median and upper quartile (Q3). The box bounds the interquartile range (IQR = Q3−Q1) with the whiskers denoting 1.5 × IQR.

In breast cancer many studies have reported on features derived from clinicopathological, DNA and/or RNA sequencing data and more recently digital pathology which may be associated with response to neoadjuvant therapy^15,17,27,28^. In the HER2 + TCHL WXS cohort here, we first individually assessed which clinicopathological and genomic features extracted from WXS data generated from pre-treatment tumour biopsies, may be associated with therapy response (Fig. 5a, Supplementary Table 8). When testing each feature individually, there was no statistically significant association between categorical clinicopathological features and future neoadjuvant therapy response (Fishers Exact test *P* > 0.05) (Fig. 5b–d). However, we noted consistent with previous reports^27,29,30^ patients who had residual disease in response to treatment tended to be younger (median age at diagnosis 45 years), ER+ (~69% vs ~31% ER+ vs ER-neg; Fishers Exact test *P* = 0.192) and have larger tumours (75% vs 25% T Stage II (>2–5 cm) vs T Stage I (< = 2 cm); Fishers exact test *P* = 0.268) (Fig. 5b–d). From mutation data, there was no significant association between TP53 (*P* = 0.379) or PIK3CA (*P* = 0.264) somatic coding mutation status and neoadjuvant therapy response (Fig. 5e, f; Fishers exact test). Again, we noted however, 46% versus 20% of patients with a somatic coding mutation in TP53 had residual disease compared to those with a pCR (Fig. 5e; Fishers exact test *P* = 0.379). Whereas 8% versus 33% of patients with a somatic coding mutation in PIK3CA had residual disease compared to those with a pCR (Fig. 5e; Fishers exact test *P* = 0.264). Overall coding tumour mutational burden (TMB) (Fig. 5e; Wilcox Test *P* = 0.076) and the fraction of the genome which is copy number altered was greater in pre-treatment tumours with residual disease (Fig. 5f; Wilcox test *P* = 0.012). We observed increased homologous recombination deficiency (HRD) copy number derived scores in pre-treatment tumour biopsies of patients with residual disease compared to those who had a pCR (Fig. 5f; Wilcox test *P* = 0.042). T cell fraction was calculated from WXS data for each patient matched tumour-normal sample using T Cell ExTRECT. WXS TCRA T cell fraction was significantly decreased in pre-treatment tumour biopsies compared to matched normal sample for both pCR (*P* = 0.032) and RD (*P* = 0.00048) cases (Fig. 5g; Paired Wilcox test). Interestingly, although not statistically significant, consistent with previous reports we observed decreased T cell fraction in pre-treatment biopsies from patients who had residual disease compared to those who had a pCR (Fig. 5h; Wilcox test *P* = 0.17).

To assess the accuracy of the digital pathology (WXS data derived TCRA T cell fraction for estimating T cells) we first evaluated its association with previously reported^20^ histopathology-derived tumour infiltrating lymphocyte (TIL) scores from pre-treatment tumour biopsy samples (Supplementary Fig. 9, 10a). Here, matched WXS TCRA T cell fraction and TIL scores were available for 18/22 cases in the WXS TCHL Cohort with 11/18 cases having T cell, CD8 + T cell and cytotoxic lymphocyte histopathology specific scoring also available for analysis (Supplementary Fig. 9). There was a strong positive correlation between WXS derived TCRA T cell fraction and histopathology scoring of T cell (rho = 0.74, *P* = 0.0087), CD8 + T cell (rho = 0.76; *P* = 0.0071) and cytotoxic lymphocyte (rho = 0.73; *P* = 0.01) TILs (Supplementary Fig. 10b). As a further validation, when selecting the subset of cases in the WXS TCHL Cohort for which RNA-Seq from pre-treatment tumour biopsy data was also available (13 of 22 cases) (Supplementary Figs. 11, 12; Supplementary Table 15), we applied MCP-Counter to pre-treatment tumour biopsies to evaluate the enrichment of stromal and immune cell types. Whole exome sequencing TCRA T cell fraction values were positively correlated with RNA-Seq data derived enrichment scores for the T cell (rho = 0.53; *P* = 0.063), CD8 + T cell (rho = 0.79; *P* = 0.0013) and cytotoxic lymphocyte (rho = 0.69; *P* = 0.0095) tumour microenvironment cell types (Supplementary Fig. 12). This suggests level of tumour infiltrating lymphocytes and immune response may be particularly important for predicting neoadjuvant therapy response in HER2+ breast cancer tumours.

### Predicting response to neoadjuvant treatment in HER2+ specific tumour subtype using logistic regression modelling

Sammut et al., (2022) recently reported on clinical and/or tumour specific characteristics which may be associated with neoadjuvant therapy response in breast cancer^27^. For patients with HER2-negative tumours they found genomic features associated with response to chemotherapy typically correlated with proliferation, TP53 mutation, tumour mutational burden, chromosomal instability, BRCA, HRD and APOBEC associated mutational signatures. Interestingly, for patients with HER2+ tumours treated with combination anti-HER2 targeted therapy and chemotherapy, response was largely independent of proliferation. This suggested that features associated with neoadjuvant therapy response may differ according to HER2 status. Therefore, having individually assessed which clinicopathological or DNA sequencing derived genomic features may be associated with neoadjuvant therapy response, we next used multivariate logistic regression modelling to investigate which of these features together may be predictive of pCR specifically in the setting of HER2+ disease.

In order to build the predictive model, we utilized publicly available datasets^27^ of clinicopathological and WXS data from neoadjuvant treated HER2+ breast cancer for training (TransNEO cohort; 57 HER2+ pre-treatment tumour biopsies) (Supplementary Table 9) and independent test set (ARTemis/PBCP cohort; 18 HER2+ pre-treatment tumour biopsies; Supplementary Table 10). The HER2 + TCHL WXS Cohort presented here was used as an independent validation dataset (TCHL cohort; 22 pre-treatment tumour biopsies) (Supplementary Fig. 6; Supplementary Table 11).

**Fig. 6:**
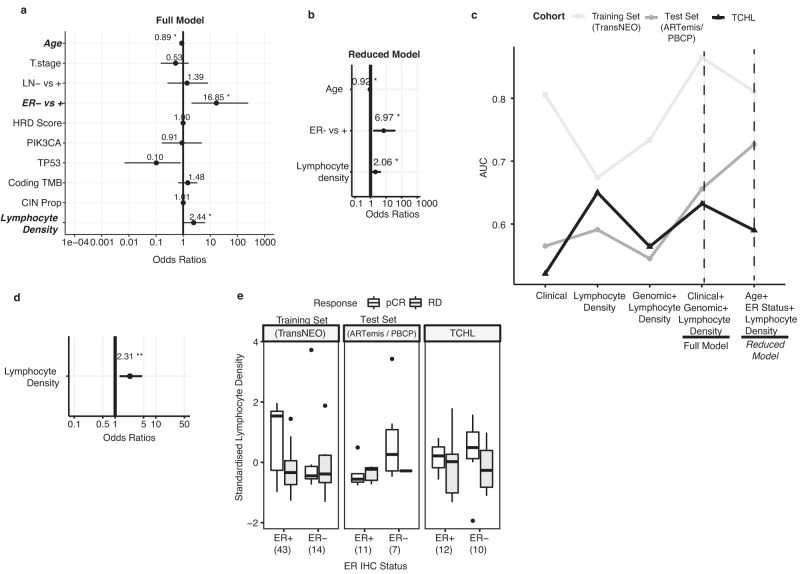
Predicting response to neoadjuvant treatment in HER2+ specific tumour subtype using logistic regression modelling. **a** Plot of multivariate analysis of associations between clinical + genomic variables+ lymphocyte density predictor variables (Full model) and pCR in the TransNEO training dataset (57 tumours). Odds ratios were calculated using multiple logistic regression model fitted using clinical and genomic features described in figure panels 5b–h. Significant associations (*P* < 0.05; logistic regression) are denoted by a bold asterisk (*). Error bars represent 95% confidence intervals. **b** Plot of multivariate analysis of associations between Age+ ER status+ lymphocyte density predictor variables in a reduced model fitted using TransNEO training dataset (57 tumours). Significant associations (*P* < 0.05; logistic regression) are denoted by a bold asterisk (*). Error bars represent 95% confidence intervals. **c** Plot of area under the curve (AUC) values for each of the five pCR predictive models (left-right) in the training set (TransNEO), test set (ARTemis/PBCP) and validation set (TCHL). Dashed lines are reference lines for the full and reduced fitted model AUCs. **d** Plot of the lymphocyte density simple logistic regression model odds ratio values in the TransNEO cohort. Significant association (*P* < 0.01; logistic regression) is denoted by a bold double asterisk (**). Error bars represent 95% confidence intervals. **e** Boxplots of standardized lymphocyte density value distribution stratified by ER status and tumour response status (pCR, RD) in the TransNEO, ARTemis/PBCP and TCHL HER2+ breast cancer cohorts. Horizontal lines in the box plots denote the lower quartile (Q1), median and upper quartile (Q3). The box bounds the interquartile range (IQR = Q3−Q1) with the whiskers denoting 1.5 × IQR.

In a similar approach to Sammut et al., (2022), a series of five pCR prediction logistic regression models were trained (Supplementary Table 12) using different combinations of clinicopathological, genomic and tumour infiltrating lymphocyte associated features which included: (1) Clinical, (2) lymphocyte density, (3) Genomic+lymphocyte density, (4) a full model (Clinical+Genomic+lymphocyte density) (Fig. 6a) and lastly (5) a reduced model (Age+ER status+lymphocyte density) which used only those predictor variables that were statistically significant (*P* < 0.05) in the full model (Fig. 6b). In the external independent test set (ARTemis/PBCP cohort), the area under the curve (AUC) for each predictive model were as follow: 0.565 (Clinical), 0.591 (lymphocyte density alone), 0.545 (Genomic+lymphocyte density), 0.656 (Full model), 0.727 (Reduced model (Age+ER status + lymphocyte density) (Fig. 6c; Supplementary Table 13). In the validation set (HER2 + TCHL WXS cohort) the AUCs were: 0.521 (Clinical), 0.65 (lymphocyte density alone), 0.709 (Genomic+lymphocyte density), 0.632 (Full model), 0.59 (Reduced model (Age+ER status + lymphocyte density)) (Fig. 6c; Supplementary Table 13). Overall, the best performing models in the ARTemis/PBCP test set were the Full (Clinical+Genomic+Lymphocyte density) and Reduced (Age+ER status+lymphocyte density) predictive models. However, in the TCHL validation set, the Full and Reduced models performed moderately well with the best performing model being the Genomic+Lymphocyte density model.

Consistent with our previous observation (Fig. 5h) regarding higher T cell fraction in HER2+ cases which had pCR compared to those with RD, we found that increasing lymphocyte density in the TransNEO cohort of 57 HER2+ pre-treatment tumour biopsies were significantly associated with increased pCR rate (Fig. 6d; Simple logistic regression, Odds Ratio: 2.3, 95% C.I: 1.28–4.61; *P* = 0.008). When stratifying standardized lymphocyte density in the TransNEO and ARTemis/PBCP cohort and T cell fraction in the TCHL cohort by ER status, we noted in the TransNEO training set cohort, lymphocyte density was particularly high for ER+ tumours which had attained a pCR (Fig. 6e).

### Clonal tumour evolution in neoadjuvant treated HER2+ tumours

**Fig. 7:**
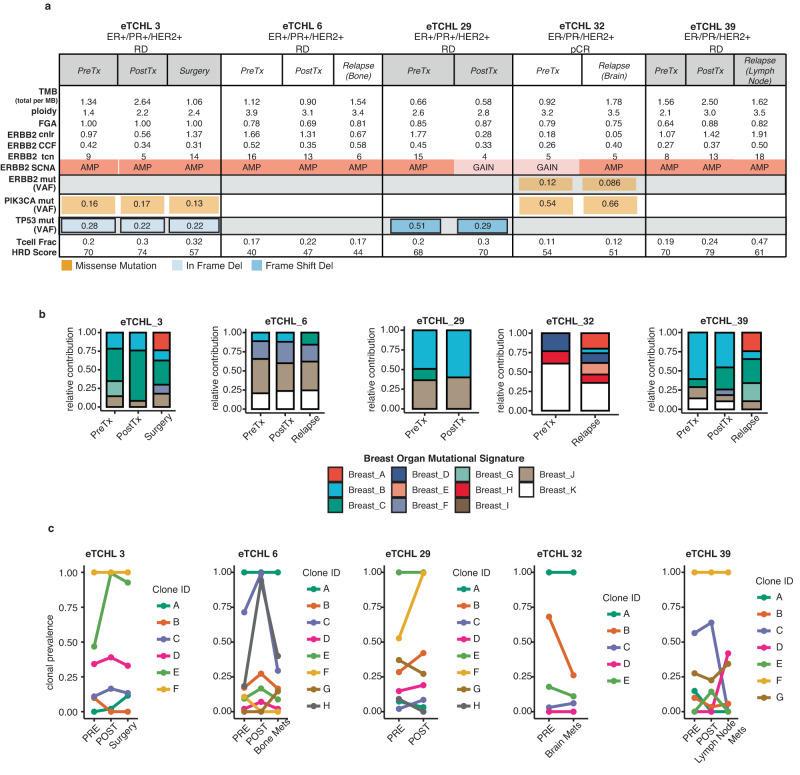
Clonal tumour evolution in matched tumour samples taken at multiple timepoints during treatment for 5 of 22 HER2+ breast cancer cases. **a** Summary from top-bottom of TMB, ploidy, FGA, ERBB2 (HER2) copy number log ratio (cnlr), cancer cell fraction (ccf), discrete total copy number (tcn) values, somatic copy number alteration (SCNA) type, ERBB2 (HER2), PIK3CA, TP53 mutation respectively if present with associated variant allele fraction (VAF) value, T cell fraction and HRD score for each matched tumour sample taken at multiple timepoints (pre-treatment, post cycle 1 (day 20) neoadjuvant treatment, surgery or relapse if occurred for 5 of 22 breast cancer cases from the HER2 + WXS cohort. **b** Stacked barchart of the relative contribution [0–1] of breast cancer specific reference mutational signatures (Breast A-K) detected in longitudinal patient matched samples (*n* = 13) (left-right). **c** Clonal prevalence line plots for each tumour clone detected using PyCloneVI for patient-matched tumour samples (*n* = 13) taken at multiple timepoints during treatment for 5 of 22 HER2+ breast cancer cases (left-right).

Tumour evolutionary analysis was carried out for 5/22 cases for which higher depth WXS was available from samples taken at multiple timepoints during the course of treatment. All five cases profiled had a poor response to neoadjuvant therapy, with 4 of 5 cases having residual disease and the remaining case being first assessed as having a pCR but subsequently developed brain metastatic disease (Fig. 7a). Overall, the mutational signature profiles of pre-treatment tumours were similar in composition to patient-matched Post Cycle 1 treatment tumour biopsy and/or subsequently collected tumours samples taken either at surgery or from metastatic disease (Fig. 7b). PyCloneVI was used to infer clonal population structure from copy number and purity adjusted variant allele frequencies (VAFs) of somatic mutations detected in longitudinally collected tumour samples for each of the five cases (Fig. 7c). The number of clones detected in each case were: 6 clones (A-F) in Case #3 (ER+), 8 clones (A-H) in Case #6 (ER+), 8 clones (A-H) in Case #29 (ER+), 5 clones (A-E) in Case #32 (ER−) and 7 clones (A-G) in Case #39 (ER−) (Fig. 7c). For each case, one clone detected in the pre-treatment tumour biopsy stably maintained a clonal prevalence value of 1.0 (i.e. all cancer cells harbour the mutations) across all timepoints, with each containing known HER2+ breast cancer specific driver genes (Supplementary Table 14). For example, the following mutations were identified as clonal in Case #3 and Case #29: PIK3CA and TP53; in Case #6, Case #29, Case #32: AURKA, FAT3 or PRKDC. Interestingly, we detected a mutation in RING-type E3 ubiquitin ligase (RNF43), a gene which is reported to negatively regulate WNT signaling in cancer present in clonal clusters across all cases and timepoints (Supplementary Table 8).

Clonal dynamics varied for all other subclones in each case with a clonal prevalence less than 1.0. We observed subclones present in pre-treatment biopsies which may harbour drug persistor somatic mutations associated with therapy response. For example, in Case #39, the clonal prevalence of the subclone labelled *G*, containing mutations in FGFR1, KMT2D, MYC driver genes, was ~0.27 in the pre-treatment tumour biopsy, decreased to ~0.22 in post cycle 1 treatment tumour biopsy but increased to ~0.34 in the lymph node biopsy (Fig. 7c). In Case #32, the subclone labelled *B* containing mutations in FAT3 and KMT2C, was present at a high clonal prevalence of ~0.68 in the pre-treatment biopsy and persisted in the brain metastatic tumour at a clonal prevalence of ~0.26. We found for some dynamic clones, such as Case #3, subclone *E* (Clonal prevalence of ~0.46 in PreTx to ~0.99 in PostTx) and Case #29, subclone *F* (Clonal prevalence of ~0.53 in PreTx to ~0.99 in PostTx) they did not contain known HER2+ breast cancer driver gene mutations but potentially novel mutations which may be selected for under the pressure of therapy. Taken together, HER2+ breast tumours are composed of (sub)clonal clusters of mutations which are established in therapy naïve tumours, maintained through treatment and may be associated with poor neoadjuvant therapy response.

## Discussion

Despite the advances in the treatment of HER2+ breast cancer, up to ~30% of patients with early stage disease may not have a pathological complete response to neoadjuvant therapy. Predictive biomarkers of neoadjuvant therapy response are needed to better identify patients with early stage disease who may benefit from tailored treatment in the adjuvant setting. Here, through comprehensive characterisation of genomic alterations and T cell fraction in pre-treatment tumour biopsies along with annotated clinicopathological characteristics we identified a subset of these features which may be associated with response to neoadjuvant therapy for HER2+ breast cancer patients. Many studies have reported that across all tumour subtypes in breast cancer, tumours with higher levels of tumour infiltrating lymphocytes are associated with increased pCR rate^17,20,27,31–33^. In agreement with these studies, we found the number of tumour infiltrating lymphocytes and/or T cell fraction present in pre-treatment tumour biopsies as an important feature of HER2+ tumours which attained a pCR. In a multivariate logistic regression model of pCR, age at diagnosis, ER status and a standardised measure of immune cell involvement either from digital pathology derived lymphocytic density or T cell fraction extracted from whole exome sequencing were found to be particularly important at predicting response. In terms of model accuracy, the area under the ROC curve value for this model in the TransNEO cohort training set was 0.82, 0.727 in the test dataset (ARTemis/PBCP Cohort) and 0.59 in the validation set (TCHL Cohort). Data from the TCHL Cohort were collected in the period before the update on standard of care regimen in the neoadjuvant setting where dual blockade with pertuzumab is now used. Currently lapatinib is not recommended for use in the neoadjuvant setting. Additional studies will need to be conducted in larger patient cohorts where standard treatment regimens are used to robustly assess model performance. Despite this, we believe we have some evidence here that our findings from the logistic regression modelling may be generalised to current standard treatment approach. The datasets (TransNEO and ARTemis/PBCP cohorts) utilised for training and testing the logistic regression model were from early stage HER2+ patient cohorts who had received chemotherapy in combination with Trastuzumab +/− Pertuzumab in the neoadjuvant setting. As such, regardless of which anti-HER2 regimen used, we believe that biomarkers of future therapy response such as age, ER status and level of tumour infiltrating lymphocytes may generalise to standard dual blockade regimens.

Many studies have highlighted the significance of the immune response in bolstering a good response to neoadjuvant therapy in early stage HER2+ breast cancer. However, the sources of greater immunogenicity in pre-treatment tumour biopsies associated with pCR is still largely unexplored in HER2+ tumours. Few have identified which specific factors are key to eliciting an anti-tumour immune response. LeSurf et al., (2017) had predicted in silico using DNA and RNA sequencing data of pre-treatment tumour biopsies from HER2+ breast cancer cases tumour neoantigen and binding affinity scores for each case^15^. They found that despite the identification of frequent TP53 and PIK3CA mutations and the production of putatively active neoantigens, there was a non-significant higher neoantigen load between pCR and RD cases. Interestingly, in this study, analysis of mutational signatures in pre-treatment tumour biopsies pointed to a greater prevalence of APOBEC associated mutational signatures in HER2+ versus HER2-neg tumours overall but furthermore the relative contribution of APOBEC associated mutational signature, Breast *C*, analogous to COSMIC reference signature 13, was highest in ER-/HER2+ compared to ER−/HER2− clinical tumour subtypes. APOBEC associated mutagenesis has been reported to be associated with increased cell proliferation and levels of tumour infiltrating lymphocytes which can elicit an anti-tumour immunogenic response^34^. Although not statistically significant, in the TCHL cohort here, we noted the median relative contribution of Breast *C* mutational signature was higher in tumours that had attained pCR compared to those that had residual disease. However, this observation did not validate in an independent cohort of pre-treatment HER2+ tumours when stratified by therapy response status.

In studies with gene expression profiling, cases assigned to the HER2-enriched (HER2-E) intrinsic molecular subtype were found to be associated with increased rates of pCR compared to the Luminal A, B, basal-like and normal-like subtypes^11,15–17,35^. An overall limitation of our study is we do not have patient-matched high quality RNA sequencing for the entire cohort. As such, due to the limited sample size we could not perform intrinsic molecular subtyping or assess if reported gene expression signatures including immune infiltration, cell proliferation, luminal differentiation and HER2 (ERBB2) gene expression^33,36^ would increase the accuracy of the predictive model. Transcriptional features derived from gene expression profiling of pre-treatment HER2+ tumour biopsies have previously been shown to influence prediction of pCR^15^. It could be possible that inclusion of intrinsic molecular subtype along with lymphocyte density and age at diagnosis may increase the predictive accuracy of the model, but also it would be interesting to further investigate the status of APOBEC associated mutational signature Breast *C* stratified by HER2-E versus other intrinsic molecular subtypes. Despite low discriminative power to predict pCR in the multivariate logistic regression, we overall observed a higher number of copy number alterations in pre-treatment tumour biopsies from patients with residual disease at surgery. Interestingly, in metastatic HER2+ breast cancer, copy number alteration burden, specifically copy number loss has been shown to be increased in rapid non responders versus exceptional responders of trastuzumab treatment^37^ and in breast cancer patients with HER2+ brain metastases^38^ suggesting that increasing levels of chromosomal instability is a particularly aggressive feature of non-responsive and treatment resistant tumours.

The status of the hormone estrogen receptor (ER) in HER2+ tumours and bi-directional ER-HER2 pathway crosstalk has repeatedly been shown to influence the clinical behaviour and therapy response of breast cancers^39^. Consistently, it has been shown that ER negative HER2+ (ER−/HER2+) breast cancers are associated with increased rates of pCR^5,7,11,15,17,35^. Here, we also observed that ER−/HER2+ tumours tended to respond better to neoadjuvant therapy with an odds ratio of ~6.97 for ER-neg versus ER+ in a predictive model of pCR along with age at diagnosis and lymphocyte density.

Altered PI3K pathway signalling is one of the most widely reported mechanisms of trastuzumab resistance in breast cancer^22,23,40^. Here, in a subset of cases which had residual disease, we noted that therapy resistance may be due to genomic aberrations in PI3K-AKT-mTOR pathway genes including PIK3CA and PTEN. We detected only PIK3CA exon 20 mutations (p.H1074R) in this cohort. Along with exon 9 mutations these are the two most frequently mutated PIK3CA mutations in breast cancer. While, overall PIK3CA mutation has been shown to be associated with decreased pCR rate in HER2+ tumours^18^, others have suggested that response may be dependent on exon 9 versus exon 20 mutations. PIK3CA exon 9 mutations have been shown to be more prevalent in cases which had residual disease which has been suggested not to be the case for exon 20 specific mutations^14,35^. Few studies have assessed if copy number gain or amplification in PIK3CA may be associated with response, however here, we observed that PIK3CA copy number gains are largely exclusive of single point mutations and may be higher in pre-treatment tumours which had residual disease at surgery.

Overall, longitudinal studies are lacking in particular for tracking subclonal expansion during the course of neoadjuvant treatment in early stage HER2+ breast cancer. The ability to deeply sequence cancer genomes has shed some light on the complexity of cancer evolution and identify subclones which may contribute to treatment response, disease progression or metastatic spread^41^. Here, for 5 of 22 cases which had a poor response to neoadjuvant treatment, clonal evolution analysis was carried out on patient-matched longitudinally collected tumour samples for which high-depth WXS data was available. Similar to others, we found pre-treatment HER2+ breast tumours contain clonally relevant driver mutations which are both important for tumour development and growth. Subclonal diversity and dynamics were evident across all cases with potential therapy resistant subclones detected containing known HER2+ breast cancer driver genes such as AURKA or novel candidate mutations such as RNF43 for future studies.

Our study has some limitations which will need to be addressed in larger cohorts in order to validate the findings described here. Firstly, as previously discussed, additional studies will need to be conducted in cohorts receiving standard treatment regimens to robustly assess model performance. Secondly, due to the limited sample size, we focused on testing differences between pCR and residual disease and were not able to further investigate these differences when stratifying by ER+ versus ER-neg, HER2+ tumour subtypes specifically.

In this study, predictive modelling of clinical and tumour specific characteristics of early-stage HER2+ breast cancer identified that age at diagnosis, ER status and level of immune cell infiltration may together be important for predicting future response to neoadjuvant therapy. The specific aspects of breast tumour biology involved in inducing an immune-rich environment to promote a good response to neoadjuvant therapy are still largely unexplored in HER2+ breast cancer. Here, we point to the possibility of APOBEC associated mutagenesis as one potential source of immunogenicity in ER-neg/HER2+ primary tumours. However, larger cohorts are needed to more comprehensively understand and validate these findings.

## Materials and methods

### Study design and patients

The design of the TCHL phase II neoadjuvant study (https://clinicaltrials.gov/ct2/show/NCT01485926; CTRIAL-IE (ICORG) 10–05) assessing TCH (Docetaxel, Carboplatin and Trastuzumab) and TCHL (Docetaxel, Carboplatin, Trastuzumab and Lapatinib) in ERBB2 (HER2) positive breast cancer patients was previously reported in detail^14^. Eligibility criteria included female breast cancer patients diagnosed with Stage T2,T3,T4a-d (TNM Staging AJCC 7th edition) or any T with lymph node positive disease tumour, HER2/neu positive (3+ by IHC or fluorescence in situ hybridisation (FISH) positive), ECOG performance status score $\le$ 1 eligible for neo-adjuvant chemotherapy and trastuzumab therapy, for which FFPE tissue was available from diagnostic biopsy and/or definitive surgical intervention. Eighty-eight patients with HER2+ breast cancer enrolled in the clinical trial were randomised to receive either neoadjuvant TCH, TCL or TCHL. As per the clinical trial protocol (NCT01485926; CTRIAL-IE (ICORG) 10–05), evaluation of response to neoadjuvant treatment was performed using tumour specimen in patients undergoing mastectomy or breast conserving procedure. Patients received trastuzumab post-operatively for 1 year. One of the secondary objectives of the trial was to identify biomarkers of anti-HER2 therapy response or resistance. As such, if available, core biopsy tumour samples were taken from patients at pre-treatment, post treatment cycle 1 (Day 20), surgery and for some patients who had progressed, tumour samples were taken from lymph node or distant metastatic disease.

### Ethics approval

In this study, we used samples that were collected under clinical trial protocols (ICORG 10–05; ClinicalTrials.gov, NCT01485926). Standardised ICORG procedures were used to acquire ethical approval for these studies. At the time of recruitment, patients were given an information leaflet and a consent form for storage and collection of biological materials, including blood and tissue samples, as well as future use of their samples for research purposes. All participants in this study had provided written informed consent. The study protocol was approved by the institutional review boards of St. James’s Hospital, Dublin; St.Vincent’s University Hospital, Dublin; Bon Secours Hospital, Cork; Cork University Hospital; Beaumont Hospital, Dublin; Mater Misericordiae University and Private Hospitals, Dublin; Galway University Hospital, Galway; Letterkenny General Hospital, Letterkenny; Mid-Western Regional Hospital, Limerick; Sligo General Hospital, Sligo; and Waterford Regional Hospital, Waterford

### Sample acquisition

Sufficient tissue for whole exome sequencing (WXS) was available from 24 normal and 39 tumour samples respectively collected from 28 of 88 patients enrolled in the TCHL phase II clinical trial. Following quality control checks, matched normal-tumour samples collected from 22 of 28 patients were included for sequencing data analysis. Tumour samples included 22 pre-treatment, 4 post-treatment cycle one (Day-20) biopsies, 1 surgical resection specimen and 3 metastatic tumours.

### Whole exome DNA sequencing

Tumour and normal samples were either snap frozen or formalin fixed paraffin embedded (FFPE). Fresh-frozen/FFPE samples were divided into three segments and a piece of tissue from the top and bottom part of each segment was sectioned, stained with haematoxylin and eosin and extensive pathological review was carried out by a trained pathologist to ensure there was a minimum of 30% tumour within the segment of the tumour samples and no tumour present in the normal samples. DNA was extracted from the tumour and normal samples using an AllPrep DNA mini kit (Qiagen, Hilden, Germany), and from whole blood samples using a DNA blood mini kit (Qiagen), according to the manufacturers protocol. DNA was quantified by Qubit fluorometer (Invitrogen, Carlsbad, CA, USA) and DNA integrity was examined by agarose gel electrophoresis. For each tumour and matched normal tissue or blood sample, exome capture was performed on sheared genomic DNA using the SeqCap EZ Library SR from Nimblegene. Paired end sequencing was carried out in two batches, first by VIB Belgium on an Illumina HiSeq2500 with a further subset of samples (Case #3, #6, #12, #29, #32, #39 and #45) selected for high depth sequencing (92X average for normal; ~280X average for tumour) by BGI Genomics (Hong Kong) on an Illumina HiSeq XTEN.

### Sequence alignment and pre-processing

The quality of raw sequencing read FASTQ files was determined using FastQC, and adapter and primer sequences and low quality 3′ end reads were cleaned using Trimmomatic (v.0.27) (https://github.com/usadellab/Trimmomatic) with the following parameters “Phred 33 LEADING:20 TRAILING:20 SLIDINGWINDOW:4:20 MINLEN:36” to remove adapter sequencing and trim low base quality calls. Sequencing reads were mapped to the human reference genome (hg38/GRCh38) using the Burrows-Wheeler Aligner (bwa mem v.0.7.5a-r405) using default parameters. According to the GATK4 best practise pipeline^42^, read duplicates were marked using Picard (v.1.1118). De-duplicated read alignments sorted using samtools (v1.5) were next processed by base quality score recalibration (BQSR) with the following references supplied with the “--known-sites” option: dbsnp_146.hg38.vcf.gz, Mills_and_1000G_gold_standard.indels.hg38.vcf.gz, 1000G_phase1.snps.high_confidence.vcf.gz.

### Sample relatedness check

Prior to somatic variant calling Somalier (v.0.2.13) (https://github.com/brentp/somalier) was used to calculate sample relatedness from sequencing data to ensure normal-tumour sample pairs came from the same individual.

### Somatic mutation calling

Somatic single nucleotide variants (SNVs), insertions and deletions (InDels) were called using Mutect2 (v.4.1.8)^43^ and Strelka (v. 2.9.10)^44^ respectively from matched normal and tumour pairs. Strelka was run with the --exome option (for WXS data only) and –callRegions option to restrict mutation calling to chr1–22,X,Y,M. In order to filter for false positive somatic mutation calls such as common variants and mapping artifacts, Mutect2 was run with gnomAD germline population reference and a panel of normal (PON) samples, generated using the CreateSomaticPanelOfNormals function part of the GATK4 (v.4.1.8) best practise pipeline. FFPE samples are known to contain mutational biases in the C > T/G > A transition. OxoG filter was applied through the read orientation bias model with Mutect2 to remove mutations with FFPE strand bias. GATK4 GetPileupSummaries and CalculateContamination was used with a set number of known germline common variants reported in ExAC at a population minor allele frequency >0.05 to calculate cross sample contamination. FilterMutectCalls was run using default parameters. Filtered Mutect2 and Strelka somatic variant calls were combined into one vcf using GATK3 (v.3.8.1) CombineVariants. Bcftools (v.1.12) (http://samtools.github.io/bcftools/bcftools.html) *norm* function was used to left align and normalise InDels. Variants passing quality control were annotated using MSK vcf2maf (https://github.com/mskcc/vcf2maf) and variant effect predictor (VEP v.96) using GRCh38, which outputs both a .vcf and .maf file format. Annotated maf files were used by MAFTools^45^ for downstream somatic mutation analysis, with annotated .vcf used as input for mutational signature analysis.

### Estimation of tumour mutational burden

Tumour mutational burden (TMB) is defined here as the number of somatic mutations per megabase of exome. The mutation rate per Mb was calculated used maftools as the total number of coding variants (SNVs, indels) divided by the length of the capture in megabases (50 Mb).

### Mutation clonal evolution analysis

PyClone-VI (v.0.1.1)^46^ was used to infer the clonal population structure within longitudinally collected patient matched tumour samples for which high depth sequencing was performed. To prepare PyClone-VI compatible input files, filtered PASS only mutations present in all matched tumour samples for one patient were concatenated together use bcftools merge to generate a “master” VCF to guide force calling of alleles with REF and ALT allele counts using GATK4 Mutect2 (--alleles flag) in each tumour sample for that patient. Variant allele frequency data was integrated with allele specific copy number calls and tumour purity values from FACETS using FACETS Suite based on the McGranahan et al., methodology^47^ for estimating the cancer cell fraction (CCF) for each mutation. Copy number and purity adjusted mutations with a major copy number >0 were clustered using PyClone-VI with the following parameters: maximum of 40 clusters, using the beta binomial probability density distribution for allele counts, performing 10 random restarts with 10,000 max iterations. Clonal prevalence was calculated at each time point by taking the median cellular prevalence value for each mutation cluster (clone).

### Somatic copy number calling approach

Somatic copy number calling was first performed using FACETS^48^ (method described in detail below). For tumour samples flagged as noisy or low purity or where ERBB2 copy number state was ambiguous (3/30 tumour samples), CNVKit^49^ (method described below) was used to independently re-interrogate copy number calls (Supplementary Fig. 1a). Taking a set of known breast cancer driver genes including ERBB2 (HER2), CNVKit was used to cross validate FACETS inferred copy number calls (Supplementary Fig. 1b). Specifically for any discordant FACETS-CNVkit copy number calls in ERBB2 (HER2) gene, the integrated genome viewer (IGV) was used to manually review the read coverage in FACETS derived segmentation for chr17 around the ERBB2 (HER2) amplicon (Supplementary Fig. 2) and re-assign copy number status if needed based on this assessment.

### Allele specific DNA copy number inference using FACETS

Total and allele-specific copy number states were inferred for all tumour samples using FACETS Suite (v 2.0.8) and FACETS (v.0.6.1) (https://github.com/mskcc/facets-suite). Tumour and matched normal bam files were pre-processed using snp-pileup (v.0.6.1) with parameters –q20 –Q20 –P100 –r25,0. A two pass implementation of FACETS using snp pileup files as input, was utilised were a low sensitivity run (cval = 150) first infers the purity and log-ratio related to diploidy, as per methodology^50^. A second higher sensitivity run (cval = 25) to detect focal events, determines the copy number state of each gene. Classification of copy number were as follow: Amplification (AMP) total copy number (TCN) $\ge$9; Gain $\ge$3 TCN $\le$8; Deletion (DEL) TCN = 0; Loss TCN = 1.

### Somatic copy number calling using CNVkit

Somatic copy number calling was additionally performed using CNVkit (v.0.9.9) (https://github.com/etal/cnvkit) on matched tumour-normal bam files. Calling was performed in exome capture regions excluding regions with low coverage or known to be inaccessible/problematic for sequencing including centromeres, telomeres and other highly repetitive regions. A pooled normal reference was generated from all normal sample bam files. Log2 copy number ratio calls were rescaled using sample specific tumour cell fraction (tumour purity estimate inference by FACETS) and normal ploidy values with simple rounding applied to generate integer value copy number calls.

### Analysis of copy number derived genomic features

Three genomic scar scores were calculated from allele specific copy number calls in FACETS: (1) fraction of chromosome which contains loss of heterozygosity (LOH), (2) Large state transitions (LST), (3) Number telomeric allele imbalance (ntAI) events.

Inference of the fraction of genome altered (FGA) by copy number alteration (CNA) was calculated for each tumour sample using the calculate_fraction_cna() function as part of the FACETS Suite (v 2.0.8) R package. For each tumour sample, the size of the chromosomal segments that deviate from the diploid copy number estimate is divided by the total size of all segments where diploid segment length is adjusted for whole genome duplication (WGD) events or not.

### Calculation of T cell fraction from WXS data

The R package for T cell ExTRECT^51^ (https://github.com/McGranahanLab/TcellExTRECT) was used to calculate the TCRA T cell fraction from whole exome sequencing of matched normal and tumour samples. Pre-defined TCRA gene segments in the tcra_seg_hg38 data file was used to extract coverage values using the runTcellExTRECT()function.

### Mutational signatures

Somatic point mutations (filtered sequencing read depth (DP) > 10) from matched normal-tumour mutation calling (Intersection set for Mutect2 & Strelka filtered (PASS only) calls) were used as input for mutational signature analysis. Note for Case #12 the pre-treatment tumour biopsy sample contained 100 or fewer substitutions and so for more optimal signature analysis the Mutect2 & Strelka filtered (PASS only; DP > 20) call set was utilised instead. The Signal^52^ version 2 (https://signal.mutationalsignatures.com/analyse2) framework was used for mutational signature extraction and signature fitting. Somatic single base substitutions were categorised by their trinucleotide context to generate a 96-channel mutational profiles. Regions of clustered substitutions i.e., kaetegis regions were filtered. The SignatureFit algorithm was run with the following parameters: GRCh38 human genome reference, breast originating organ, 250 bootstraps, threshold 1%, *p*-value < 0.05. To assess the robustness of mutational signature detection when using WXS data, the deconstructSigs R package^53^ and exome trimer count normalisation method was applied to the same set of somatic mutation catalogues used as input to Signal framework. Extracted signatures were fitted to COSMIC reference signatures (version 2 (March 2015); https://cancer.sanger.ac.uk/signatures/signatures_v2/).

### Publicly available datasets for external validation and analysis of genomic alterations

In order to compile a list of HER2+ subtype specific known breast cancer driver genes somatic copy number alterations, SNV and InDels were cross referenced to a list of HER2+ subtype specific breast cancer driver genes previously reported in two studies^54,55^ to be frequently altered in HER2+ breast cancer. Rinaldi et al., (2020) had reported on the frequency of somatic genomic alterations stratified by breast cancer subtype including HER2+ from analysis of targeted sequencing of ~11,000 unmatched primary breast, local recurrence and distant metastatic tumours using the FoundationOne assay^54^. Smith et al., (2021) had performed genomic profiling of 733 HER2-amplified breast tumours from 664 patients with HER2+ metastatic breast cancer^55^. Those genes reported in the study to have a somatic mutation frequency >1% were combined with the Rinaldi et al., (2020) geneset to compile a list of known driver genes that were frequently altered in HER2+ breast cancer. ComplexHeatmap R package^56^ was used to visualize co-occurrence and frequency of SCNA and SNVs in pre-neoadjuvant treatment tumour biopsies.

For analysis of mutational signature profiles, the Degasperi et al., (2020) breast organ specific mutational signature profiles (Breast *A-K*) from breast tumours originally sequenced as part of the Nik Zainal et al., (2016) study of 560 primary breast cancer genomes^26^ was used. Supplementary Table 1 from the Nik Zainal study (included as Supplementary Table 16 here) detailed clinico-pathological information for all 560 breast cancer cases and was used to (a) assign a clinical subtype based on ER,PR,HER2 status of the primary tumour, (b) identify which cases were HER2+ subtype (73/560) and (c) were annotated as having a tumour sample that was removed pre-treatment (24/73). These identifiers were then cross referenced against the Degasperi et al., (2020) (Supplementary Table 17) mutational signature profiles to retrieve a table of patients for whom breast organ specific mutational signature profiling had been performed (438/560). 68 of 438 cases were HER2+ subtype specific with 19 of 68 annotated as having their tumour sample removed pre-treatment. Two additional publicly available independent WXS breast cancer cohorts with annotated neoadjuvant therapy tumour response status (pCR or RD) were utilized for analysis of COSMIC reference mutational signature profiles (Signature 1–30) in pre-treatment tumour biopsies from patients with breast cancer who received neoadjuvant therapy: LeSurf et al., (2017)^15^ and Sammut et al., (2022)^27^ comprised of 48 and 168 breast cancer cases respectively. Briefly, from the LeSurf research article, Supplementary Table S1 and S8 containing pathologic response information and deconstructSigs derived mutational signature profiles were downloaded and imported into R for visualisation. From the Sammut et al., (2022)^27^ study, clinical and sample information was downloaded from Supplementary Table 1. deconstructSigs derived mutational signature profiles were downloaded from supplementary data available at https://github.com/cclab-brca/neoadjuvant-therapy-response-predictor.git.

### Estimation of tumour microenvironment cell fraction

FastQC was used to assess quality control metrics for single end sequencing reads (FASTQ) from RNA sequencing data. BBDuk from the BBMap toolkit (https://sourceforge.net/projects/bbmap/) was used for sequencing adapter removal and read trimming. Two-pass read alignment to the GRCh37 human reference transcriptome was performed using STAR (https://github.com/alexdobin/STAR) followed by read duplicate marking and removal using Picard. Read counting was carried out using the featureCounts function from the Subread (https://subread.sourceforge.net/). Read count data was normalised using transcripts per million (TPM) method. The MCPcounter package (v1.2.0) in R was used to run the Microenvironment cell population (MCP) counter method^57^ using normalised gene expression data from RNA sequencing of 13 pre-treatment tumour biopsy samples from patients in the TCHL Cohort here.

### Logistic regression modelling

The glm() function as part of the stats R package was used to fit a multiple logistic regression model where the binary response variable was neoadjuvant therapy tumour response status (1 = pCR, 0 = RD). Residual disease (RD) category was composed of those cases which were classified as having either partial or no response. pROC and caret R packages were used for testing the model and generating area under the curve (AUC) values.

A multiple logistic regression model was trained using clinicopathological (Age at diagnosis, Tumour (T) Stage, ER IHC status, Lymph node (LN) status at diagnosis), genomic (HRD Score, PIK3CA, TP53 somatic mutation status, Coding TMB (per 45.5 Mb), chromosomal instability (CIN)) and digital pathology (lymphocyte density) feature data generated as part of the Sammut et al., (2022) TransNEO study^27^. This dataset included genomic profiling of 57 HER2+ subtype pre-treatment (chemotherapy+HER2 targeted therapy) biopsies of breast tumours (155 of 168 cases of breast cancer which had more than 1 cycle of neoadjuvant therapy). As part of the Sammut et al., study an external validation dataset had been generated comprised of 75 patients (18/75 HER2+ subtype) treated with neoadjuvant therapy recruited for the ARTemis trial or Personalised Breast Cancer (PBCP) study. Here, the TransNEO and ARTemis/PBCP specific datasets were utilised for training and test datasets respectively for multiple logistic regression modelling. As per the Sammut et al., methodology only cases that had received at least one cycle of neoadjuvant chemotherapy and one cycle of anti-HER2 therapy (if HER2+) were used to model which features are associated with response to neoadjuvant therapy. The previously described HER2 + TCHL WXS Cohort clinicopathological and genomic data derived feature were used as a validation dataset for model testing. Standard normal (*z*-score) scaling was applied to TCRA T cell fraction values prior to model testing.

### Statistics and reproducibility

Statistical analyses were performed using R version 4.1.2. All statistical tests (paired Wilcoxon Rank Sum (Mann-Whitney *U*-test), Wald etc) and their associated *P* values are two-sided, with a *P* value < 0.05 considered to be statistically significant unless otherwise stated. Reported *q* values represent Benjamini-Hochberg corrected *P* values. Logistic regression models: odds ratios (ORs) with 95% confidence intervals (CIs) and Wald *P* values with no correction for multiple testing applied are presented. Any predictor variables with a Variance inflation factor (VIF) value > 5 were further investigated for multicollinearity. R package jtools (v.2.2.0) and sjPlot (v. 2.8.11) were used to summarise and plot logistic regression model output.

### Reporting summary

Further information on research design is available in the Nature Research Reporting Summary linked to this article.

### Supplementary information

Supplementary Information

Supplementary Tables 1–17

Reporting Summary

## Data Availability

All summary data supporting the findings of this study are available within the article and/or its supplementary materials. For WXS data from the 22 HER2+ breast cancer cases (matched normal, pre-treatment primary breast tumour biopsy +/− post-treatment (Cycle 1 (day20) +/− surgically resected primary breast tumour or distant metastatic tumour samples) and RNA sequencing data for 13 pre-treatment tumour biopsy samples the processed files are available on figshare [10.6084/m9.figshare.22708834]. Raw DNA whole exome sequencing (WXS) data for the tumour-normal sample pairs and RNA-Seq for tumour samples collected under clinical trial protocols (ICORG 10–05; ClinicalTrials.gov, NCT01485926) will be made available upon request and under regulatory compliance via data usage agreement (DUA). Please contact the corresponding author with data access requests. Tumour infiltrating lymphocyte (TIL) histopathology scores for TCHL Cohort from the Eustace et al., (2021) publication is available from [10.1007/s10549-021-06244-1]. Supplementary Table 1 from Rinaldi et al., (2020) targeted sequencing study of approx. 11,000 unmatched primary breast, local recurrence and distant metastatic tumours using the FoundationOne assay is available at [10.1371/journal.pone.0231999]. Supplementary Tables 1 and 8 from LeSurf et al., (2017) is available at [10.1093/annonc/mdx048]. For logistic regression models: training and test set data is available for download from Sammut et al., (2022) [https://github.com/cclab-brca/neoadjuvant-therapy-response-predictor]. Breast organ specific mutational signature profile data from Degasperi et al., (2020) is available from [10.1038/s43018-020-0027-5] and included here as Supplementary Tables16 and 17.
