# Supplementary material for: Predictive modelling of response to neoadjuvant therapy in HER2+ breast cancer
Source: NPJ Breast Cancer. 2023 Sep 27;9:72. doi: 10.1038/s41523-023-00572-9 (PMC10533568; doi:10.1038/s41523-023-00572-9)
Supplement: Supplementary file 1 — Supplementary Information [file 41523_2023_572_MOESM1_ESM.pdf]

a

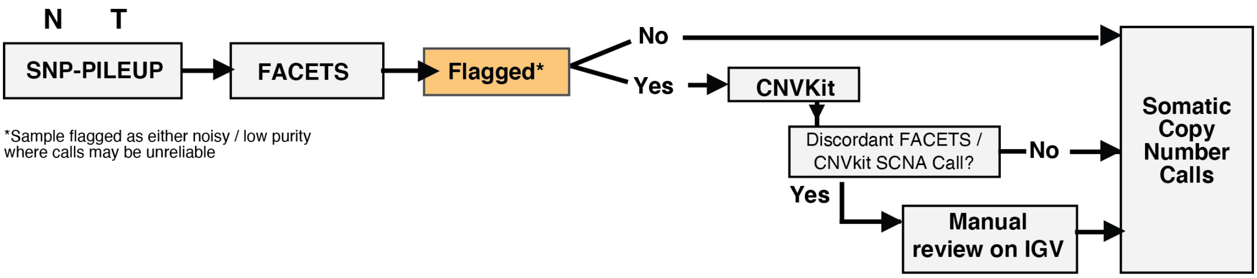

b

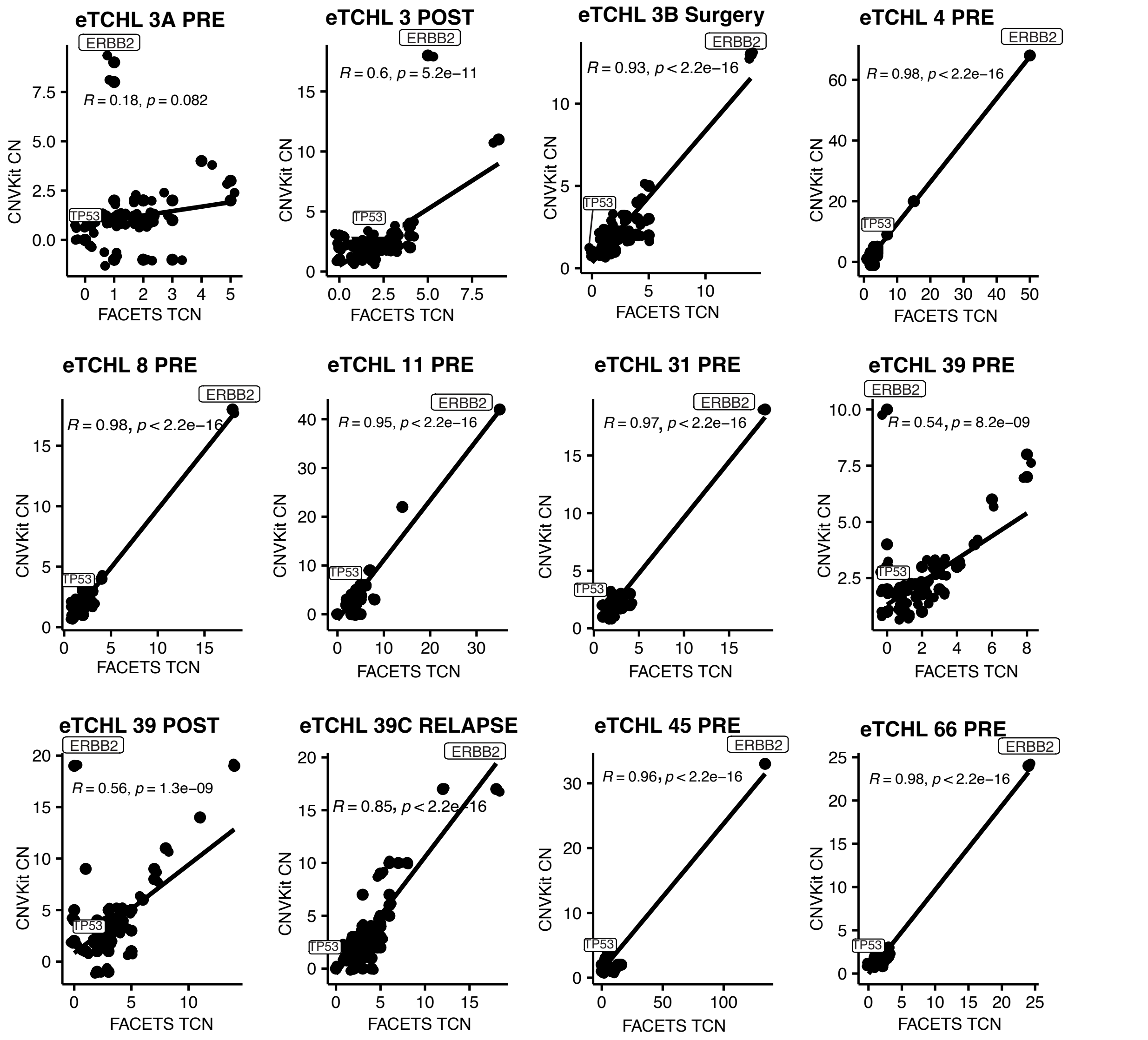

Supplementary Figure 1. Two step FACETS-CNVKit somatic copy number calling approach. (a) Graphical summary of the two-step FACETS-CNVKit somatic copy number calling approach **(b)** Scatterplots and corresponding Spearman correlation coefficient (*R*) quantify the relationship between CNVKit derived discrete copy number (CN) call (y-axis) and FACETS derived total copy number (TCN) call (x-axis) for a set of known breast cancer driver genes including ERBB2 (HER2) (labelled) for tumour samples (from left-right, top-bottom) flagged using the somatic copy number calling approach described in (a). Spearman Rho Rank correlation statistical test $P<0.05$.

ERBB2(HER2) chr17

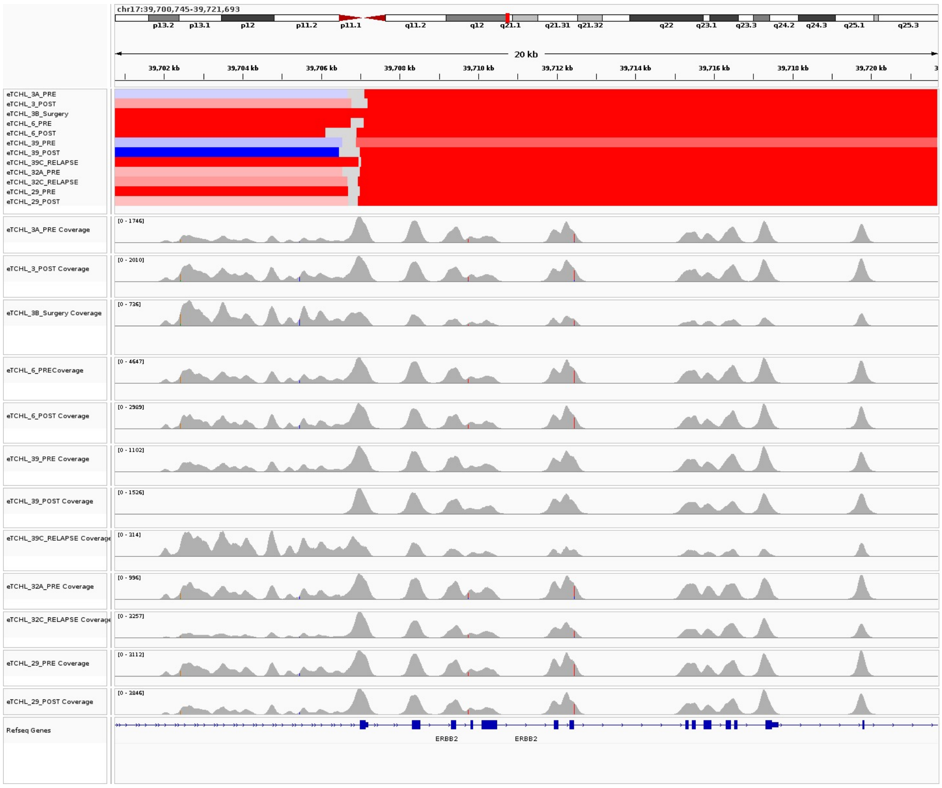

MYC chr8

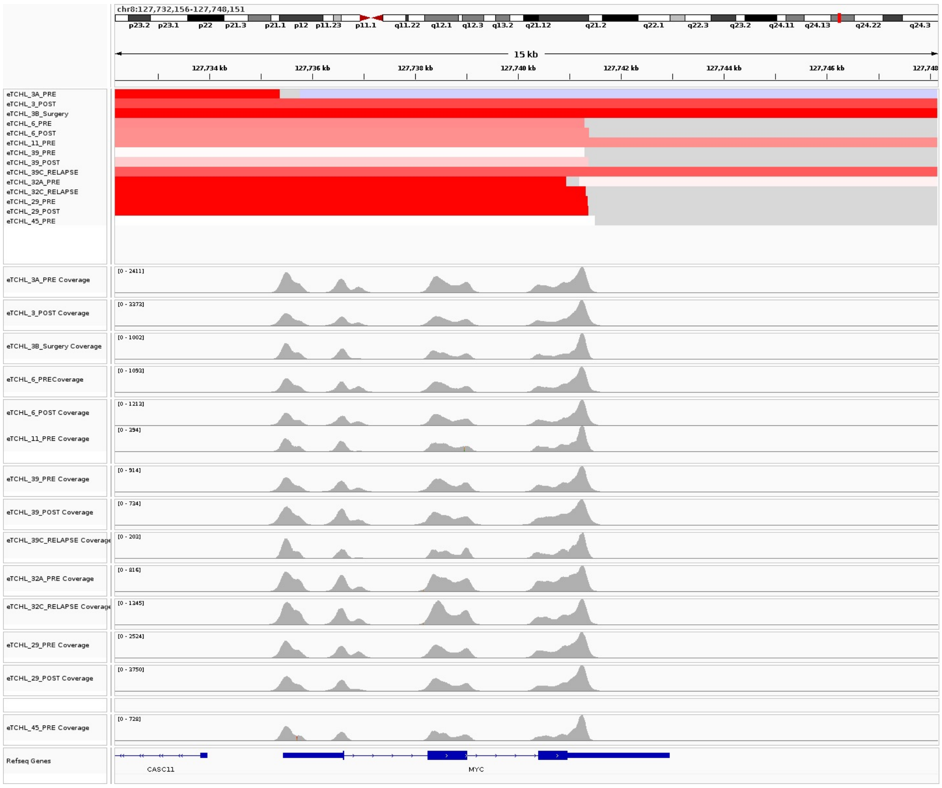

Supplementary Figure 2. Manual review of somatic copy number calls using IGV. From the Integrated Genome Viewer (IGV) FACETS derived medium copy number log ratio value (Top panel; red: copy number gain or amplification while blue : copy number loss or deletion for the segment) from joint segmentation of total and allele specific copy number calling in the ERRB2 (HER2) gene on chromosome 17 and MYC oncogene on chromosome 8 for flagged tumour samples. Bottom panel shows the sequencing coverage over captured DNA regions including exons for the ERBB2 (HER2) and MYC oncogenes. IGV Reference human genome track GRCh38.

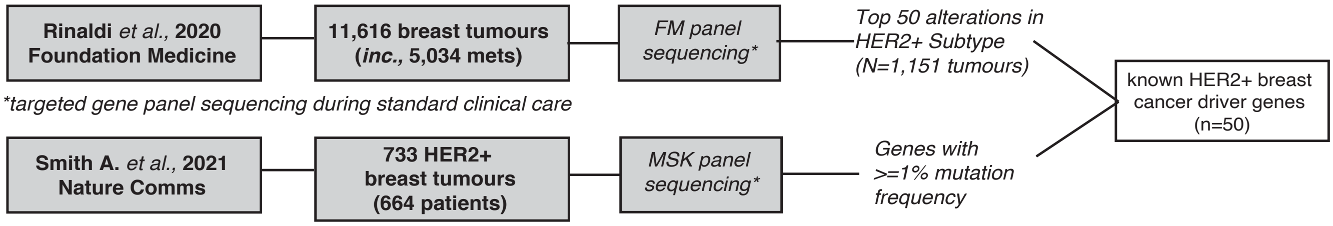

Supplementary Figure 3. HER2+ subtype specific known breast cancer driver gene list. Graphical schematic shows the approach used (from left to right) to generate a list of known breast cancer driver genes frequently altered in HER2+ tumour subtype from previously published breast cancer genomic datasets (Rinaldi *et al.,* (2020) and Smith *et al.,* (2021)) for whole exome sequencing data analysis.

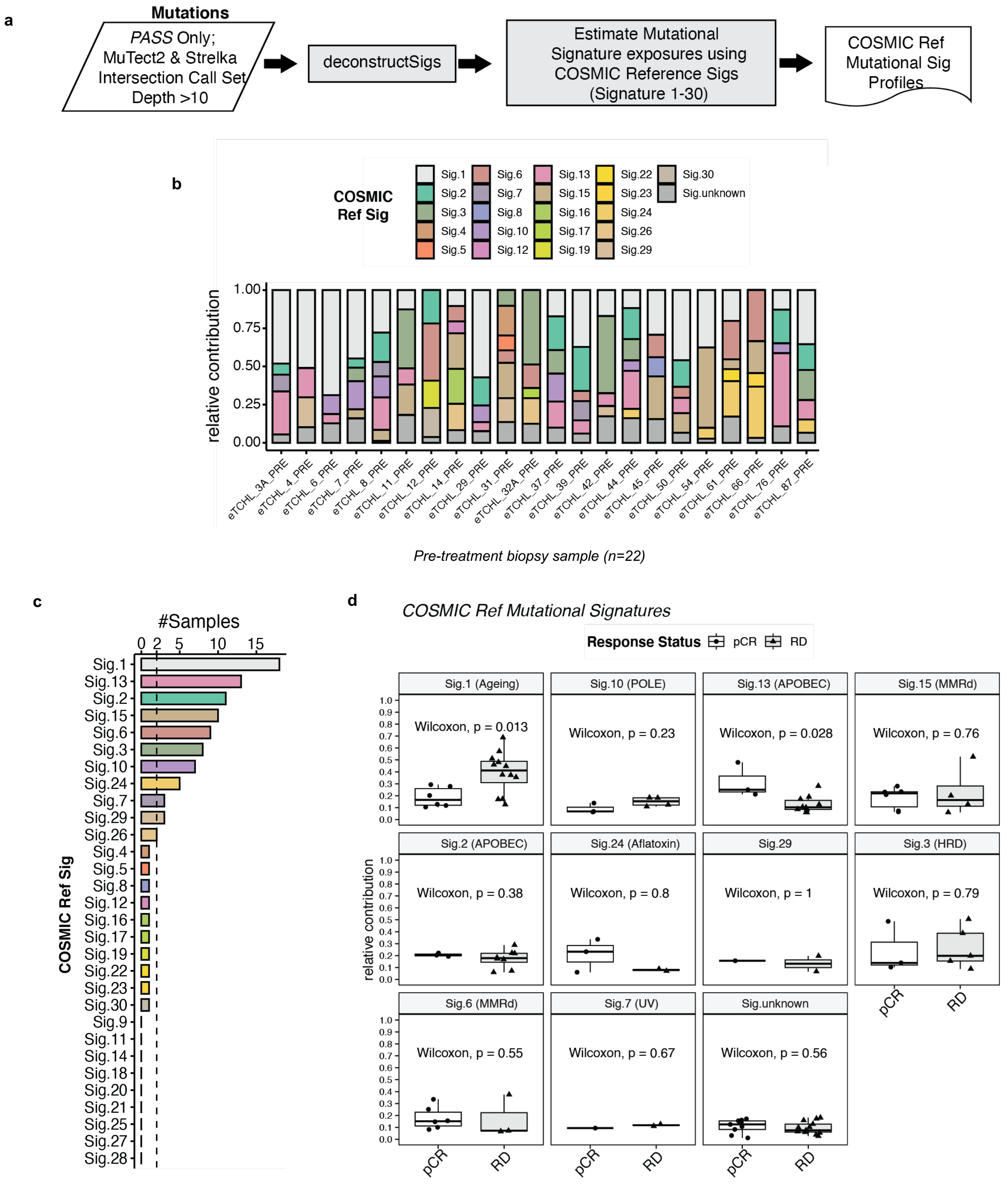

Supplementary Figure 4. Mutational signature profiling using deconstructSigs. (a) Overview of mutational signature workflow using deconstructSigs. **(b)** Stacked barchart of the relative contribution [0-1] of COSMIC reference mutational signatures detected in each pre-treatment tumour biopsy sample (n=22) (left-right) from the HER2+ TCHL WXS cohort. **(c)** Barchart shows frequency of COSMIC reference mutational signatures (top-bottom) across all pre-treatment biopsy samples (n=22). Dashed line indicates 2 or more samples. **(e)** Boxplots show the distribution of relative contribution values of frequent COSMIC reference signatures (present in >2 samples) in pCR (n=9; white) compared to RD (n=13; grey) classified tumours (Wilcox Test P<0.05). Horizontal lines in the box plots denote the lower quartile (Q1), median and upper quartile (Q3). The box bounds the interquartile range (IQR = Q3 − Q1) with the whiskers denoting 1.5 x IQR.

**Dataset**	**# breast tumour samples**	**# Annotated as HER2+ clinical subtype**	**# pre-treatment tumour samples**	**Mutational signature fitting applied by original publication**
Nik Zainal *et al.,* (2016)	560	73	24	COSMIC RefSig
Degaspieri, Nik Zainal *et al.,* (2020)	438 / 560	68	19 / 24	Breast Organ Specific
LeSurf, Mardis *et al.,* (2017)	48	48	48	COSMIC RefSig
Sammut, Caldas *et al.,* (2022)	168	65	65	COSMIC RefSig

Supplementary Figure 5. Publicly available genomic datasets utilised for external validation and analysis of genomic alterations. Table summary of publicly available genomic datasets utilised in this study to externally validate and analyse genomic alterations including mutational signature profiles found in breast tumours. Table includes per dataset: total number of breast tumour samples sequenced, the number of these samples which are annotated as HER2+ clinical tumour subtype, the number of these HER2+ subtype cases which had pre-treatment tumour sample sequenced and lastly if either COSMIC reference signature (Signature 1-30) or Signal breast organ specific (Breast *A-K*) mutational signature fitting was applied to mutational signatures extracted from analysis of somatic SNVs.

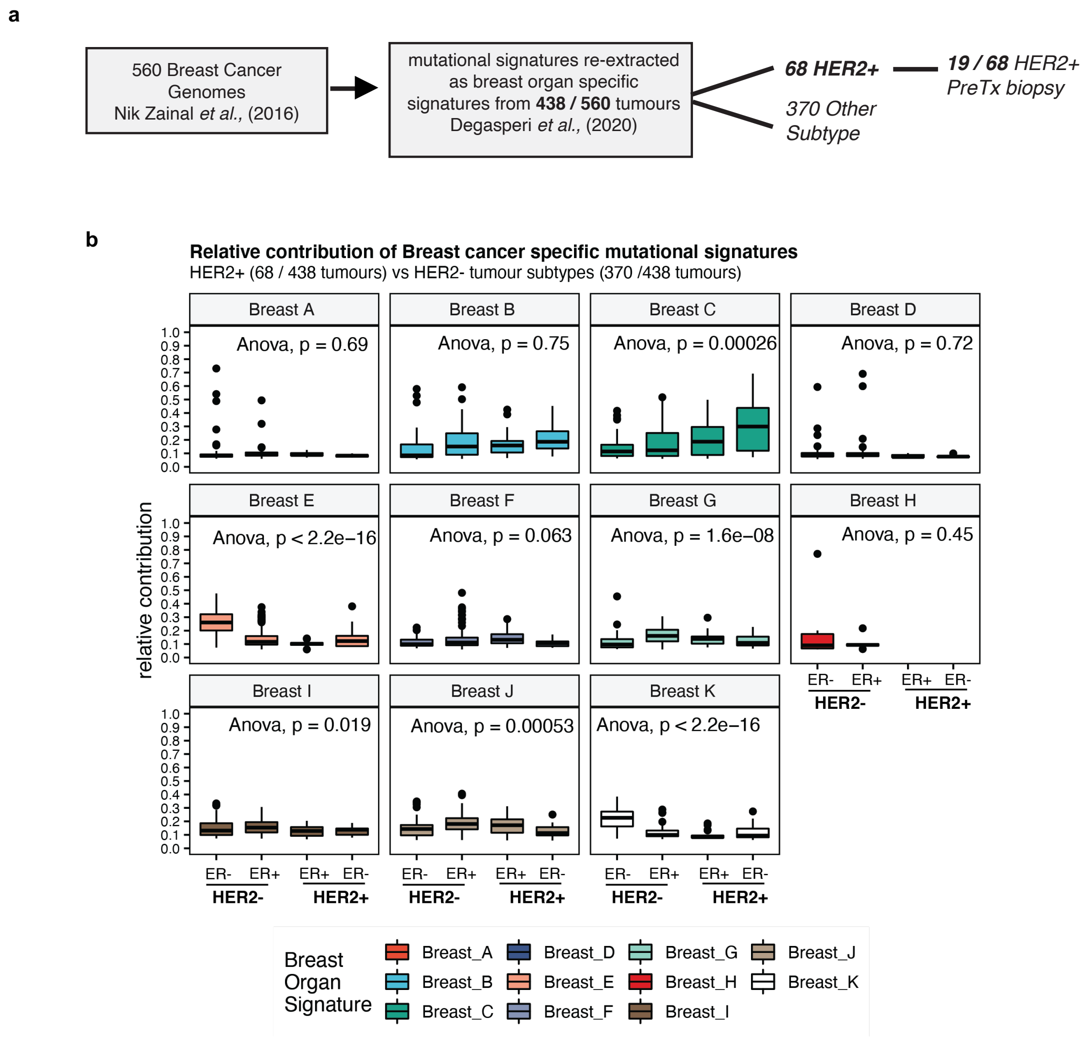

Supplementary Figure 6. Analysis of breast organ specific mutational signatures in HER2+ versus HER2-negative breast cancer genomes. (a) Graphical overview of selection of breast cancer cases annotated by clinical subtype from the Nik Zainal *et al.,* (2016) 560 breast cancer genome study for which breast organ specific mutational profiles had been re-extracted from 438 of 560 tumours in a later study by Degasperi *et al.,* (2020) **(b)** Boxplots of the relative contribution of each breast organ specific mutational signature (Breast *A-K*) stratified by clinical subtype (from left-right in each Breast mutational signature panel: ER-/HER2-, ER+/HER2- (370 of 438 total tumours) and ER+/HER2+, ER-/HER2+ (68 of 438 total tumours)). One-way ANOVA test (*P*<0.05). *P*-value text annotated on each panel. Horizontal lines in the box plots denote the lower quartile (Q1), median and upper quartile (Q3). The box bounds the interquartile range (IQR = Q3 − Q1) with the whiskers denoting 1.5 x IQR.

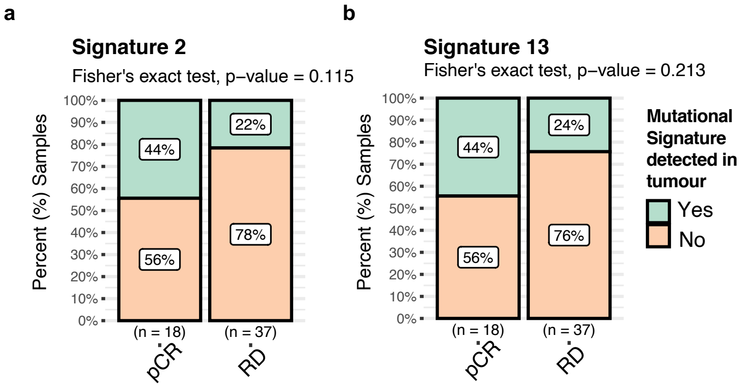

Supplementary Figure 7. Proportion of HER2+ cases annotated by APOBEC associated mutational signature status according to tumour response status. (a) Stacked barchart shows the proportion of patients with HER2+ breast cancer who had a pathological complete response (pCR; N=18) or residual disease (RD; N=37) at surgery following neoadjuvant treatment in the Sammut *et al.,* (2022) cohort. For pCR and RD cases respectively, the percentage of cases (%) is specific according to APOBEC associated COSMIC Reference Signature 2 status (Yes (light green) mutational signature detected in tumour; No (light orange) signature not detected in tumour). **(b)** Same as (a) but for COSMIC Reference Signature 13.

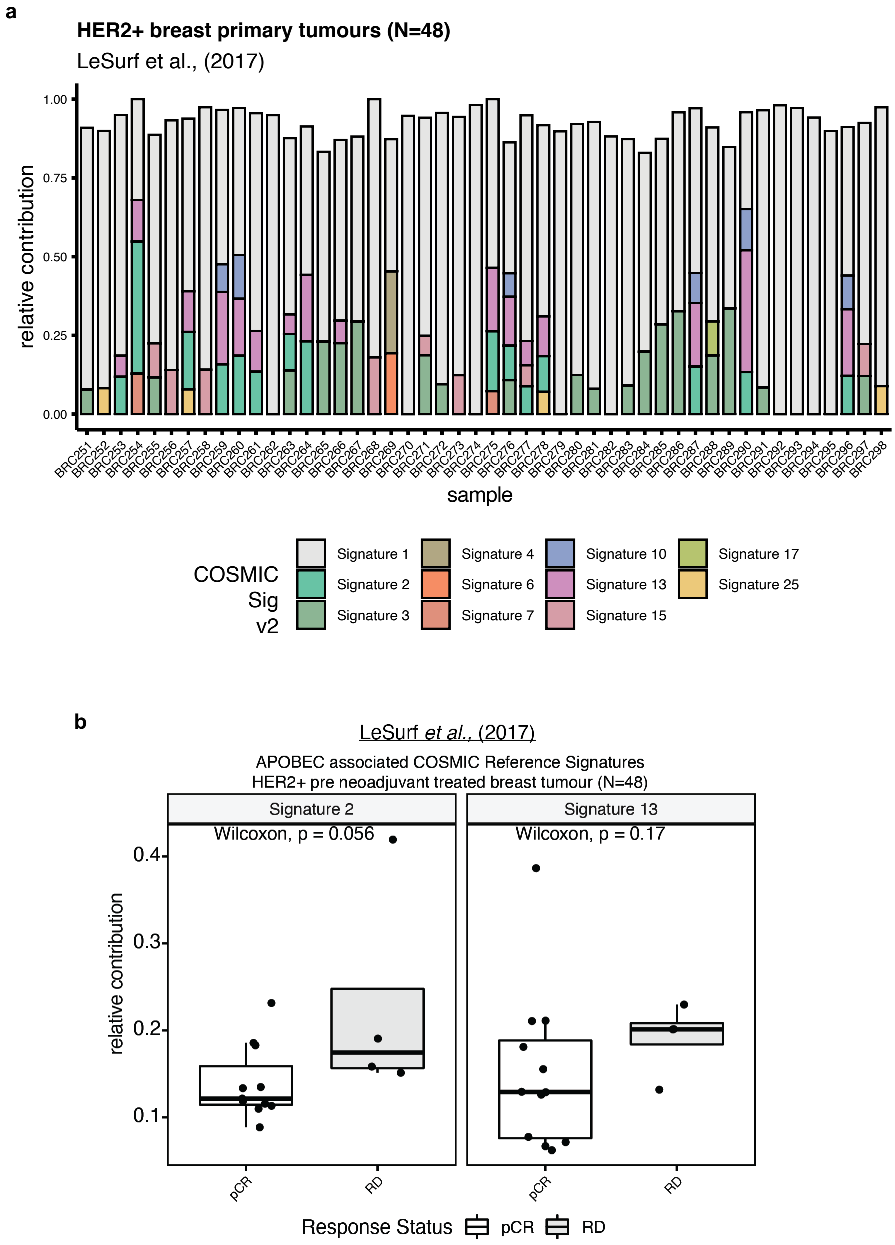

Supplementary Figure 8. COSMIC reference mutational signature profiles in pre-treatment tumour samples from the LeSurf *et al.,* (2017) HER2+ breast cancer cohort. (a) Stacked barchart of the relative contribution [0-1] of COSMIC reference (version 2) mutational signature profiles detected in each pre-treatment biopsy tumour sample (left-right) from LeSurf *et al.,* (2017) HER2+ breast cancer cohort. **(b)** Boxplots show distribution of APOBEC associated mutational signature values (COSMIC Signature 2 and Signature 13) if present, stratified by tumour response status (pCR vs RD) in the LeSurf *et al.,* (2017) HER2+ breast cancer cohort (Wilcox Test, P>0.05). Horizontal lines in the box plots denote the lower quartile (Q1), median and upper quartile (Q3). The box bounds the interquartile range (IQR = Q3 − Q1) with the whiskers denoting 1.5 x IQR.

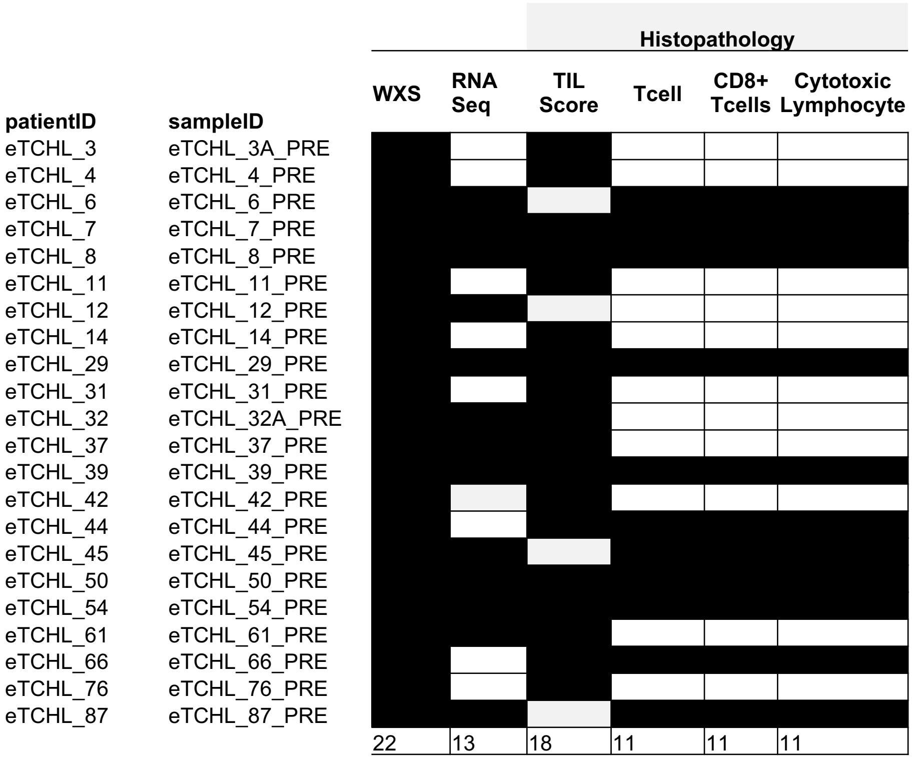

Supplementary Figure 9. Summary of matched sequencing and TIL histopathology data availability for TCHL WXS Cohort. Tileplot summarises for each pretreatment tumour biopsy sample collected from patients in the TCHL WXS Cohort here the availability of WXS (DNA), RNA sequencing data and/or tumour infiltrating lymphocyte (TIL) and T cell specific scores from histopathology. Black square denotes sample was available for sequencing or histopathology analysis. White square denotes sample was unavailable.

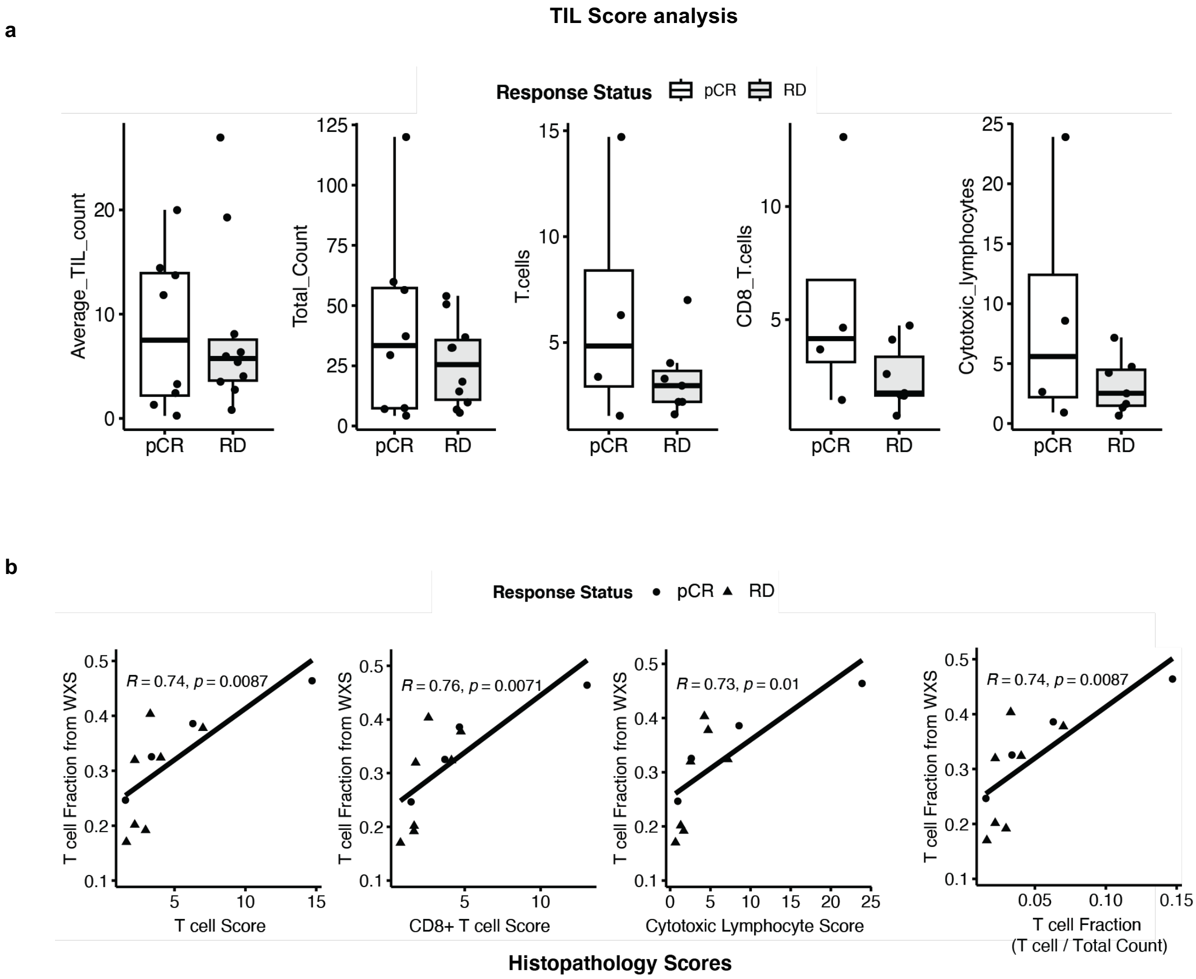

Supplementary Figure 10. Association of histopathology TIL score with WXS TCRA T cell fraction (a) Boxplots of histopathology scores for (from left to right) Average TIL count, Total Count, T cell, CD8+ T cell and Cytotoxic Lymphocyte from pre-treatment tumour biopsy samples **(d)** Scatterplots and corresponding Spearman correlation coefficient (rho (R)) quantify the relationship between TCRA.T cell fraction values derived from WXS data using T cell ExTRECT method versus (from left to right) T cell, CD8+ T cell and Cytotoxic Lymphocyte histopathology scores. Horizontal lines in the box plots denote the lower quartile (Q1), median and upper quartile (Q3). The box bounds the interquartile range (IQR = Q3 − Q1) with the whiskers denoting 1.5 x IQR. Spearman Rho Rank correlation statistical test *P*<0.05.

RNA Sequencing

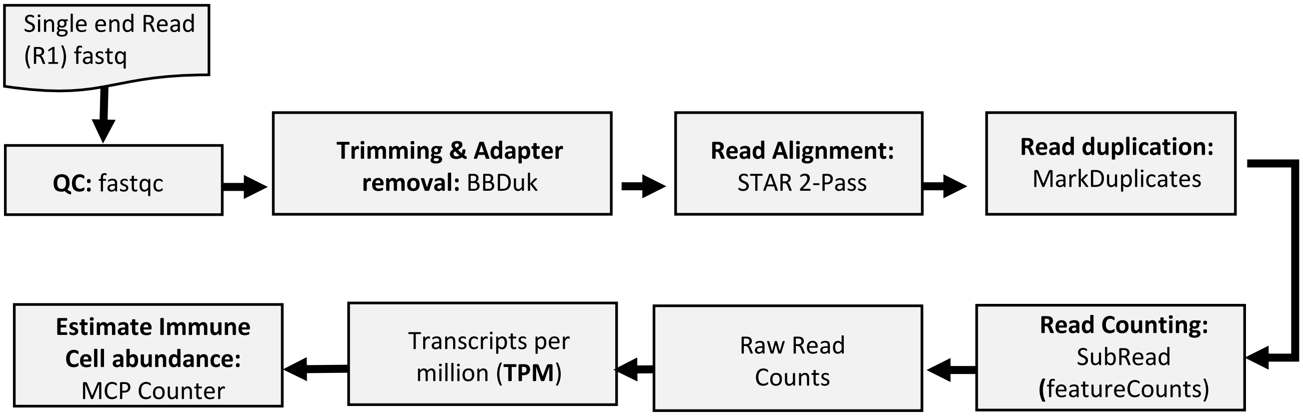

Supplementary Figure 11. RNA sequencing data processing for estimating immune cell abundance in pre-treatment tumour samples. Graphical schematic shows the approach used (from left to right; top to bottom) to process RNA sequencing data generated from pre-treatment tumour biopsy samples to estimate immune cell abundance using MCP Counter.

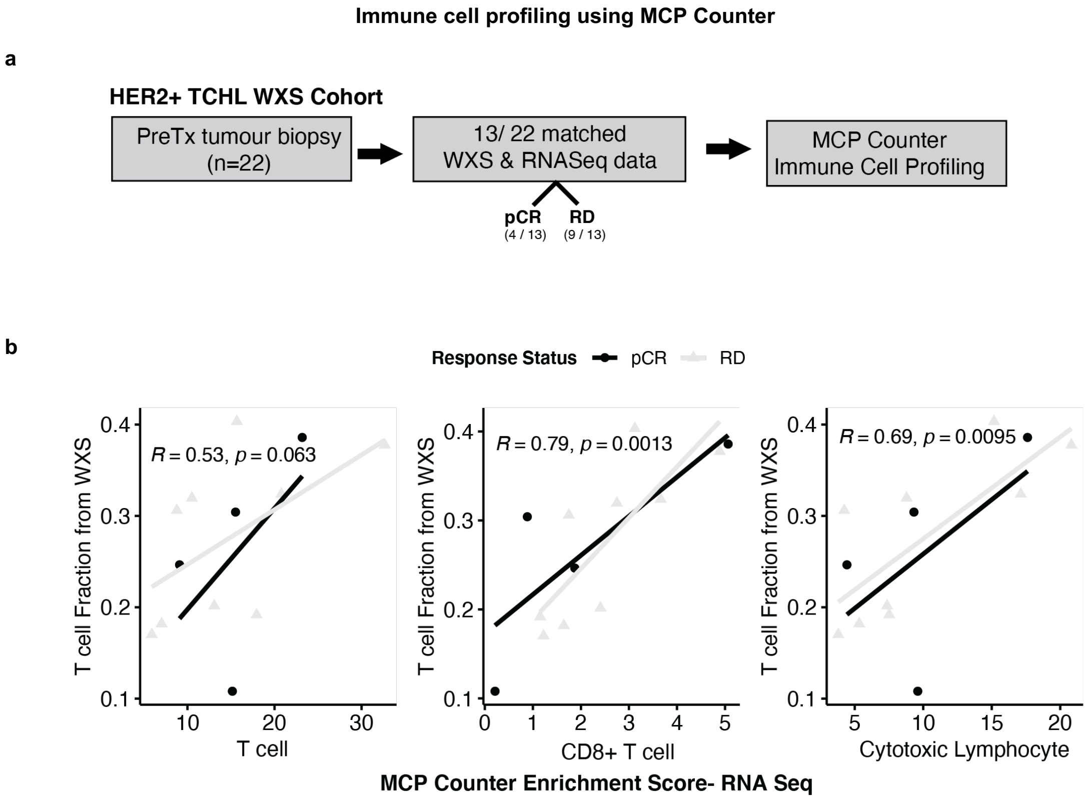

Supplementary Figure 12. Estimation of tumour microenvironment cell enrichment using gene expression data and MCP-Counter method. (a) Overview of the number of pre-treatment tumour biopsy samples with RNASeq matched with WXS data for MCP-Counter immune cell profiling. **(b)** Scatterplots and corresponding Spearman correlation coefficient (R) quantify the relationship between T cell fraction values derived from WXS data using T cell ExTRECT method versus (from left to right) T cell, CD8+ T cell and Cytotoxic Lymphocyte enrichment score values estimated from RNASeq data using MCP-Counter method. Spearman Rho Rank correlation statistical test $P<0.05$.
