# Supplementary material for: Predictive modelling of response to neoadjuvant therapy in HER2+ breast cancer
Source: NPJ Breast Cancer. 2023 Sep 27;9:72. doi: 10.1038/s41523-023-00572-9 (PMC10533568; doi:10.1038/s41523-023-00572-9)
Supplement: Supplementary file 3 — Reporting Summary [file 41523_2023_572_MOESM3_ESM.pdf]

nature portfolio

Corresponding author(s): Simon J Furney, Bryan T Hennessy

Last updated by author(s): Jul 13, 2023

Reporting Summary

Nature Portfolio wishes to improve the reproducibility of the work that we publish. This form provides structure for consistency and transparency in reporting. For further information on Nature Portfolio policies, see our Editorial Policies and the Editorial Policy Checklist.

Statistics

For all statistical analyses, confirm that the following items are present in the figure legend, table legend, main text, or Methods section.

n/a	Confirmed	
☐	☒	The exact sample size (*n*) for each experimental group/condition, given as a discrete number and unit of measurement
☐	☒	A statement on whether measurements were taken from distinct samples or whether the same sample was measured repeatedly
☐	☒	The statistical test(s) used AND whether they are one- or two-sided<br>*Only common tests should be described solely by name; describe more complex techniques in the Methods section.*
☐	☒	A description of all covariates tested
☐	☒	A description of any assumptions or corrections, such as tests of normality and adjustment for multiple comparisons
☐	☒	A full description of the statistical parameters including central tendency (e.g. means) or other basic estimates (e.g. regression coefficient) AND variation (e.g. standard deviation) or associated estimates of uncertainty (e.g. confidence intervals)
☐	☒	For null hypothesis testing, the test statistic (e.g. *F*, *t*, *r*) with confidence intervals, effect sizes, degrees of freedom and *P* value noted<br>*Give P values as exact values whenever suitable.*
☒	☐	For Bayesian analysis, information on the choice of priors and Markov chain Monte Carlo settings
☒	☐	For hierarchical and complex designs, identification of the appropriate level for tests and full reporting of outcomes
☐	☒	Estimates of effect sizes (e.g. Cohen's *d*, Pearson's *r*), indicating how they were calculated

Our web collection on statistics for biologists contains articles on many of the points above.

Software and code

Policy information about availability of computer code

Data collection

DNA Seq
- hg38 / GRCh38 human reference genome
- Trimmomatic (v0.27)
- bwa mem (v0.7.5a-r405)
- GATK4 (v.4.2.0.0)
- Picard (v1.1118)
- Somalier (v 0.2.13)
- Mutect2 (v.4.1.8)
- Strelka (v.2.9.10)
- samtools (v.1.5)
- bcftools (v.1.12)
-GATK3 CombineVariants (v.3.8.1)
- snp-pileup v.0.6.1
- FACETS v.0.6.1
- FACETS Suite v 2.0.8
- CNVKit (v.0.9.9)
- Signal v.2.0 (https:// signal.mutationalsignatures.com/analyse)
- vcf2maf v1.6.17
- VEP version 96
- PyClone-VI (v.0.1.1)
-T cell ExTRECT

RNA Seq
- GRCh37 human reference transcripts (human_g1k_v37.fasta)
- BBDuk v.35.85
- STAR v.2.5.2a
- Picard
- MultiQC v.1.7
- SubRead v1.5.0-p3 (Homo_sapiens.GRCh37.75.saf)

Data analysis

No custom codes were used in the analysis reported in this study. All relevant references are provided in the methods section.
All data analysis using R 4.1.2
R package versions:

-ComplexHeatmap v.2.10.0
-maftools v.2.10.05
-biomaRt v.2.42.1
-ggpubr v.0.6.0
-ggplot2 v.3.3.3
-deconstructSigs v.1.8.0
-signature.tools.lib v.2.3.0
-facetsSuite v 2.0.8
-MCPcounter v.1.2.0

Other Software:
Adobe Illustrator 2023 v.27

For manuscripts utilizing custom algorithms or software that are central to the research but not yet described in published literature, software must be made available to editors and reviewers. We strongly encourage code deposition in a community repository (e.g. GitHub). See the Nature Portfolio guidelines for submitting code & software for further information.

Data

Policy information about availability of data

All manuscripts must include a data availability statement. This statement should provide the following information, where applicable:

- Accession codes, unique identifiers, or web links for publicly available datasets
- A description of any restrictions on data availability
- For clinical datasets or third party data, please ensure that the statement adheres to our policy

All relevant data is included in the article and in its Supplementary Tables. For WXS data from the 22 HER2+ breast cancer cases (matched normal, pre-treatment primary breast tumour biopsy +/- post-treatment (Cycle 1 (day20) +/- surgically resected primary breast tumour or distant metastatic tumour samples) and RNA sequencing data for 13 pre-treatment tumour biopsy samples the processed files are available on figshare [doi : 10.6084/m9.figshare.22708834]. Raw RNA and DNA sequencing data for all samples are not published openly in order to protect participant identities, but will be made available upon request and under regulatory compliance via a data usage agreement (DUA). Please contact the corresponding author with data access requests that will be granted once the DUA is signed. Tumour infiltrating lymphocyte (TIL) histopathology scores for TCHL Cohort from the Eustace et al., (2021) publication is available from [doi: 10.1007/s10549-021-06244-1]. Supplementary Table 1 from Rinaldi et al., (2020) targeted sequencing study of approx. 11,000 unmatched primary breast, local recurrence and distant metastatic tumours using the FoundationOne assay is available at [doi: 10.1371/journal.pone.0231999]. For logistic regression models: training and test set data is available for download from Sammut et al., (2022) [https://github.com/cclab-brca/neoadjuvant-therapy-response-predictor]. Breast organ specific mutational signature profile data from Degasperi et al., (2020) is available from [doi: 10.1038/s43018-020-0027-5].

Research involving human participants, their data, or biological material

Policy information about studies with human participants or human data. See also policy information about sex, gender (identity/presentation), and sexual orientation and race, ethnicity and racism.

Reporting on sex and gender

All breast cancer patients enrolled in this study were female.

Reporting on race, ethnicity, or other socially relevant groupings

We do not category patients based on race, ethnicity or other socially relevant groupings in this study.

Population characteristics

The study population involves female patients Age > 18 years, with histologically proven HER2+ breast cancer, enrolled on the phase II TCHL clinical trial for which neo-adjuvant chemotherapy and trastuzumab was considered a valid therapeutic strategy. A description of the covariate relevant study population and tumor characteristics including tumour stage, estrogen receptor status, age at diagnosis (by age ranges 30-39, 40-49, 50-59, 60-69, 70-79), anti-HER2 treatment and other clinical characteristics can be found in Supplementary Table 1.

Recruitment

Samples were sequenced from patients recruited and enrolled as per the clinical trial protocol (NCT01485926; CTRIAL-IE (ICORG) 10-05)

Ethics oversight

In this study, we used samples that were collected under clinical trial protocols (ICORG 10-05; ClinicalTrials.gov, NCT01485926). Standardised ICORG procedures were used to acquire ethical approval for these studies. At the time of recruitment, patients were given an information leaflet and a consent form for storage and collection of biological materials, including blood and tissue samples, as well as future use of their samples for research purposes. All participants in this study

had provided written informed consent. The study protocol was approved by the institutional review boards of St. James's Hospital, Dublin; St.Vincent's University Hospital, Dublin; Bon Secours Hospital, Cork; Cork University Hospital; Beaumont Hospital, Dublin; Mater Misericordiae University and Private Hospitals, Dublin; Galway University Hospital, Galway; Letterkenny General Hospital, Letterkenny; Mid-Western Regional Hospital, Limerick; Sligo General Hospital, Sligo; and Waterford Regional Hospital, Waterford

Note that full information on the approval of the study protocol must also be provided in the manuscript.

Field-specific reporting

Please select the one below that is the best fit for your research. If you are not sure, read the appropriate sections before making your selection.

☒ Life sciences ☐ Behavioural & social sciences ☐ Ecological, evolutionary & environmental sciences

For a reference copy of the document with all sections, see nature.com/documents/nr-reporting-summary-flat.pdf

Life sciences study design

All studies must disclose on these points even when the disclosure is negative.

Sample size	For sequencing, no statistical method was used to predetermine sample size. Sample size was determined by availability of pre-treatment breast tumour biopsy, post-treatment, surgically resected primary breast or metastatic tumour tissue for sequencing.
Data exclusions	24 normal and 39 tumour samples respectively were originally collected from 28 of 88 patients enrolled in the TCHL phase II clinical trial. Following quality control checks, 9 samples were excluded before WXS data analysis commenced.
Replication	For patient sequencing studies replication of each individual sample is not possible and is not utilized in standard practice.
Randomization	Sequenced samples were collected as part of the phase II TCHL clinical trial (ICORG 10-05; ClinicalTrials.gov, NCT01485926) where randomization of patients enrolled in the trial was performed. For sequencing data analysis, no further randomization was performed. Samples were already assigned to pCR and RD groups as per the clinical trial protocol (NCT01485926; CTRIAL-IE (ICORG) 10-05) where the evaluation of response to neoadjuvant treatment was performed using tumour specimen in patients undergoing mastectomy or breast conserving surgical procedure.
Blinding	During generation of tumour sequencing data and analysis no blinding was performed when allocating groups by response status.

Reporting for specific materials, systems and methods

We require information from authors about some types of materials, experimental systems and methods used in many studies. Here, indicate whether each material, system or method listed is relevant to your study. If you are not sure if a list item applies to your research, read the appropriate section before selecting a response.

Materials & experimental systems

n/a	Involved in the study	
☒	☐	Antibodies
☒	☐	Eukaryotic cell lines
☒	☐	Palaeontology and archaeology
☒	☐	Animals and other organisms
☐	☒	Clinical data
☒	☐	Dual use research of concern
☒	☐	Plants

Methods

n/a	Involved in the study	
☒	☐	ChIP-seq
☒	☐	Flow cytometry
☒	☐	MRI-based neuroimaging

Clinical data

Policy information about clinical studies
All manuscripts should comply with the ICMJE guidelines for publication of clinical research and a completed CONSORT checklist must be included with all submissions.

Clinical trial registration	ICORG 10-05; ClinicalTrials.gov, NCT01485926. Registered on 2 December 2011.
Study protocol	https://clinicaltrials.gov/study/NCT01485926
Data collection	Data was collected between the study start date of October 2010 and study completion May 2018.
Outcomes	In the clinical trial, the primary outcome measure was pathological complete response. As per the clinical trial protocol (NCT01485926; CTRIAL-IE (ICORG) 10-05), evaluation of response to neoadjuvant treatment was performed using tumour specimen

in patients undergoing mastectomy or breast conserving procedure. Response was determined as per the AJCC 7th edition.
